# Confinement of a Au–N-heterocyclic carbene in a Pd_6_L_12_ metal–organic cage†

**DOI:** 10.1039/d0ra07509d

**Published:** 2020-10-27

**Authors:** Lihua Zeng, Shujian Sun, Zhang-Wen Wei, Yu Xin, Liping Liu, Jianyong Zhang

**Affiliations:** MOE Laboratory of Polymeric Composite and Functional Materials, School of Materials Science and Engineering, School of Chemistry, Sun Yat-Sen University Guangzhou 510275 China zhjyong@mail.sysu.edu.cn

## Abstract

A Au(i)–N-heterocyclic-carbene (NHC)-edged Pd_6_L_12_ molecular metal–organic cage is assembled from a Au(i)–NHC-based bipyridyl bent ligand and Pd^2+^. The octahedral cage structure is unambiguously established by NMR, electrospray ionization-mass spectrometry and single crystal X-ray crystallography. The electrochemical behaviour was analyzed by cyclic voltammetry. The octahedral cage has a central cavity for guest binding, and is capable of encapsulating PF_6_^−^ and BF_4_^−^ anions within the cavity.

## Abstract 2

A Au(i)–NHC-edged Pd_6_L_12_ molecular cage is assembled from a Au(i)–NHC-based bipyridyl bent ligand and Pd^2+^.

## Graphical abstract

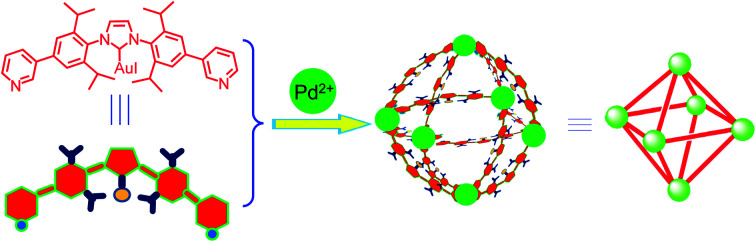

## Introduction

The self-assembly of metal–organic cages (MOCs)^1–3^ has been investigated for diverse potential applications, such as catalysis,^4^ sensing,^5,6^ molecular storage and sequestration,^7^ drug delivery,^8^ and so on. For MOCs the shape, cavity size, and ligand can influence their guest binding and transformation capability.^9–11^ N-heterocyclic-carbenes (NHCs) are a class of electron-donating ligands,^12^ and have been applied in catalyzing various organic transformations.^13,14^ Discrete assemblies based on NHC ligands including molecular metallocycles and cages have received increasing attention^15–18^ because they may induce reactivity change, selectivity and product distribution variation.^19,20^ However, few molecular cages with well-defined cavities are reported in the literature.^21–23^ Remarkably, Nitschke *et al.* reported M_4_L_6_ (M = Zn(ii), Cd(ii)) cages with Au(i)–NHC-based ligand.^23^ In our effort to build NHC-based cages with large cavity, we wish to report herein that a Pd_6_L_12_ metal–organic cage containing twelve Au(i)–NHC centres is assembled from a rigid, bent N-heterocyclic-carbene-based bispyridyl ligand and palladium(ii) ions.

## Results and discussion

**Fig. 1:**
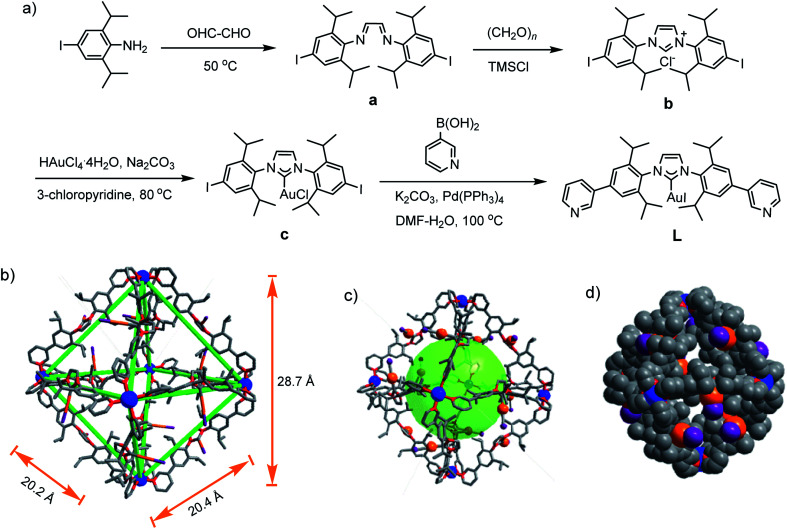
(a) Synthetic route of L, single crystal X-ray structures of Pd_6_L_12_, (b) sticks, (c) ball-and-stick model, (d) space-filling model. Anions, solvent molecules and hydrogen atoms are omitted for clarity. Color coding: C: gray; N: red; I: purple; Au: orange; Pd: blue.

**Fig. 2:**
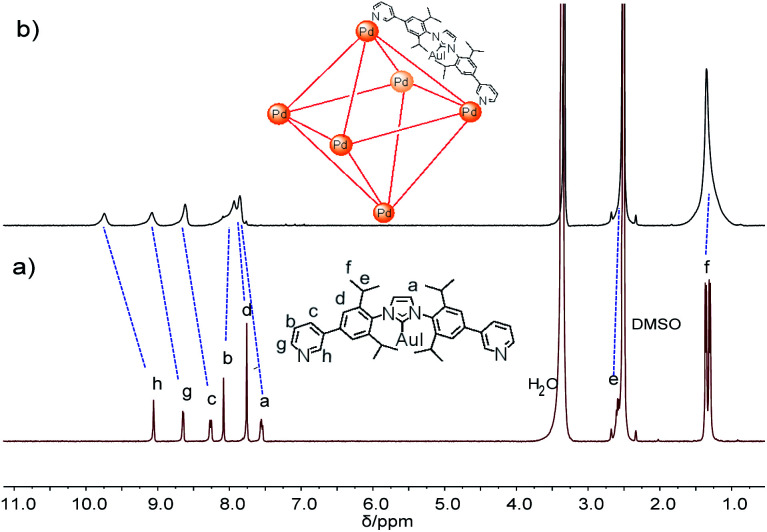
^1^H NMR spectra of (a) L and (b) Pd_6_L_12_ in DMSO-*d*_6_ (400 MHz, 298 K).

For the self-assembly of Pd_6_L_12_ cage, bis(pyridyl)-functionalised Au(i)–NHC ligand L was designed and synthesized (Fig. 1a). First diiodo-functionalised imidazolium chloride (b) was synthesized from bis-Schiff's compound a^24^ and paraformaldehyde *via* a ring-closing step in the presence of chlorotrimethylsilane. Then Au(i)–NHC compound c^25^ was synthesized by the reaction of imidazolium salt b with HAuCl_4_·4H_2_O and 3-chloropyridine in the presence of base (Na_2_CO_3_). Subsequently, L was synthesized by Suzuki–Miyaura cross-coupling reaction between c and 3-pyridylboronic acid (Fig. S1–S10†). ^1^H NMR shows that the imidazolium C–H resonance appeared at *δ* 10.34 for b and disappeared for L. ^13^C NMR spectra show the resonance of the imidazolium C–H carbon at 139.60 ppm for b and the resonance of the metallated carbene-carbon atoms at 185.55 ppm. The coordination cage Pd_6_L_12_ was successfully assembled by heating a 2 : 1 mixture of L and Pd(NO_3_)_2_·2H_2_O in DMSO at 70 °C for 6 h. ^1^H NMR indicates that the quantitative formation of Pd_6_L_12_ (12 NO_3_^−^ anions are omitted for clarity, the same hereinafter) (Fig. 2 and S11†). The cage is highly symmetric. Compared with the free ligand L the original sharp signals of pyridine moieties turn into broad peaks and shift downfield, for example the signals at 9.05, 8.64 and 8.26 ppm for the H_i_, H_h_ and H_c_ pyridyl protons are downfield-shifted to 9.74, 9.09 and 8.62 ppm, respectively, which is ascribed to the coordination of palladium(ii) ions to the ligand.

Gratifyingly, single crystals of Pd_6_L_12_ (NO_3_^−^ salt) suitable for X-ray diffraction analysis were obtained over one month by slow diffusion of ethyl acetate vapour into a solution of Pd_6_L_12_ in DMSO (Table S1†). The crystals crystallize in the trigonal space group *R*3̄. The cubic symmetric unit is composed of six Pd(ii) metal centres linked by twelve L ligands, forming the octahedron edges (Fig. 1b). Nevertheless, nitrate ions and solvent molecules could not be reasonably located in this highly disordered structure. The bend angle between pyridine rings and central Au–NHC ring is 174.1°. Two pyridyl donors on the same ligands adopt *syn*-conformation. The bond angles C_carbene_–Au–I_trans_ range from 176 to 179° are in fact close to linearity (C18–Au1–I1 176.1(11)°) and the Au1–C18 bond length of 1.96(3) Å, which is close to those of the reported (NHC)gold(i) complexes.^26^ Each Pd(ii) has a square planar geometry with Pd–N bond distances of 1.98(2)–2.03(2) Å and the angle between the two 3-pyridyl coordinating motifs in L is 88–93°, within the normal range for those reported analogous pyridine-based ligand assembled Pd_6_L_12_ complexes.^27,28^ The cavity size is approximately 20.2 × 20.4 × 28.7 Å^3^, which is defined by the six Pd(ii) ions. The opposing Pd(ii)–Pd(ii) distance is 28.7 Å and adjacent Pd–Pd distances are 20.2–20.4 Å. One of the two diisopropyl groups on each ligand points to the cavity, and the C–C distance of diisopropyl groups on opposite ligands is 16.3 Å.

**Fig. 3:**
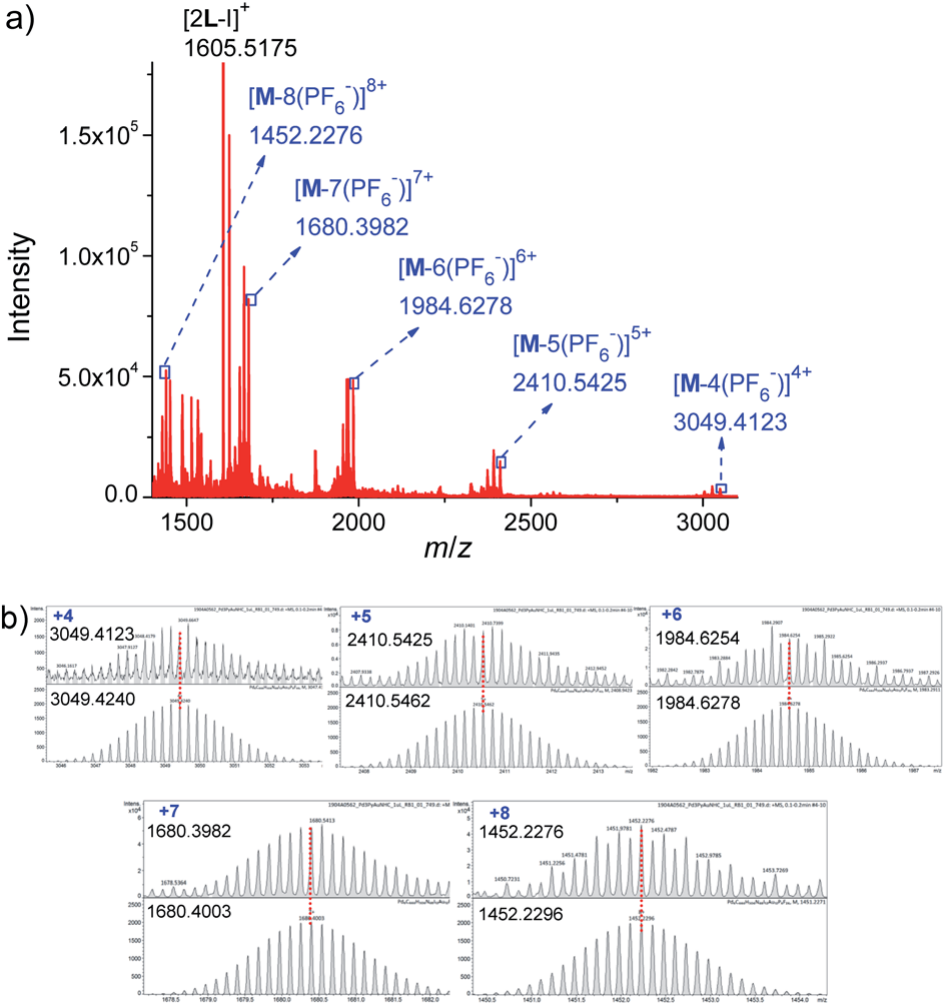
(a) ESI-TOF-MS spectrum of Pd_6_L_12_ (M) (PF_6_^−^ as counterion) and (b) measured (top) and calculated (bottom) isotope patterns for various charge states observed for [Pd_6_L_12_]^12+^·12PF_6_^−^.

Further evidence for Pd_6_L_12_ was provided by ^1^H–^1^H homonuclear correlation spectroscopy (COSY) and ^1^H–^1^H nuclear overhauser effect spectroscopy (NOESY), which both reveal important cross peaks between the two observed sets of NMR signals (*e.g.* H_c_–H_b_ and H_c_–H_h_) (Fig. S12 and S13†). In addition, diffusion-ordered NMR spectroscopy (DOSY) shows the selective formation of a single species (Fig. S14†). The same diffusion coefficient at *D* = 5.75 × 10^−11^ m^2^ s^−1^ corresponds to the dynamic radius of 19.0 Å according to the Stokes–Einstein equation.^28^ Further structural evidence was given by electrospray ionization-mass spectrometry (ESI-MS) (Fig. 3). After anion exchange of NO_3_^−^ for PF_6_^−^ ions in DMSO solution, a series of prominent peaks with continuous charge states of [M–(PF_6_^−^)_*n*_]^*n*+^ (*n* = 4–8) were detected for Pd_6_L_12_. The isotopic distribution patterns of each peak agreed well with the simulated patterns.

The chemical composition of L and Pd_6_L_12_ were characterized by Fourier transform infrared spectroscopy (FT-IR) (Fig. S15†). The absorption of Pd_6_L_12_ at 1632 cm^−1^ corresponding to the C=N in-ring stretching in the pyridine rings shifts to lower energy compare with L at 1638 cm^−1^, which is attributed to the coordination of L to Pd^2+^*via* the nitrogen atom. Both L and Pd_6_L_12_ exhibit characteristic C–N_carbene_ bands typically at 1467 and 1443 cm^−1^.^29^ The strong absorption band at approximately 1384 cm^−1^ is attributed to the NO_3_^−^ ions for Pd_6_L_12_.

**Fig. 4:**
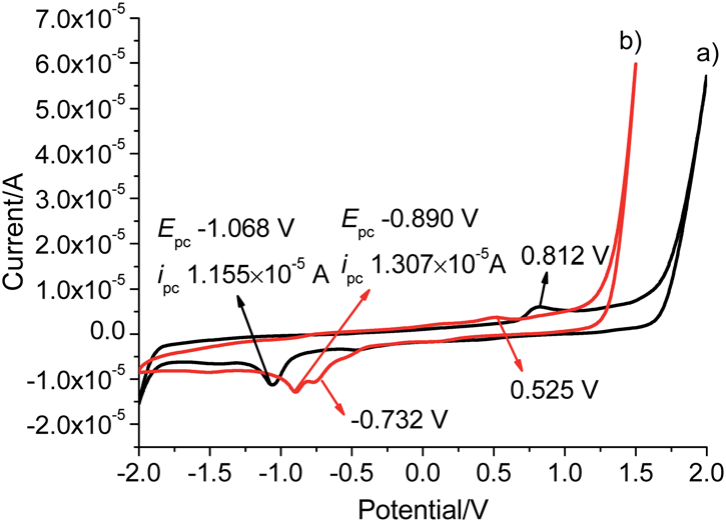
Cyclic voltammograms of (a) L and (b) Pd_6_L_12_ in DMSO solution at a scan rate of 50 mV s^−1^, results are reported *versus* Ag wire.

To investigate the electrochemical behaviour, cyclic voltammetry analyses were carried out in the potential range from −2.0 to +2.0 V at a scan rate of 50 mV s^−1^ in a solution of tetrabutylammonium hexafluorophosphate (TBAPF_6_) in dry DMSO (0.10 mol L^−1^) as a supporting electrolyte on glassy carbon electrode (3 mm in diameter) (Fig. 4). L gave a reduction wave at a potential of −1.068 V, indicating the reduction of Au(i) to metallic Au(0) corresponding to previously reported Au(i)–NHC analogues.^30,31^ The analogous process was significantly shifted to a more anodic potential at −0.890 V for Pd_6_L_12_. In addition to the reduction processes, a single irreversible oxidation peak was also observed at 0.812 V for Pd_6_L_12_, positively shifted by 0.287 V from L (+0.525 V), which is consistent with the oxidation processes of I^−^ to I_2_ on clean glassy carbon surface.^32,33^ It is worth noting that a new irreversible cathodic potential appeared at −0.732 V, attributed to the reduction process of NO_3_^−^ to NO_2_^−^ within the cage,^34^ perhaps owing to the change of ionic status in the cavity after coordination.^35^ The reduction current for Pd_6_L_12_ were acquired at scan rates of 50–200 mV s^−1^, showing that two reduction waves are located below the scan rates of 100 mV s^−1^ (Fig. S16†). With the increase in the sweep rate, the two waves merge into one broad peak, suggesting that the redox process takes place under simple diffusion-control for the cage.^36^

**Fig. 5:**
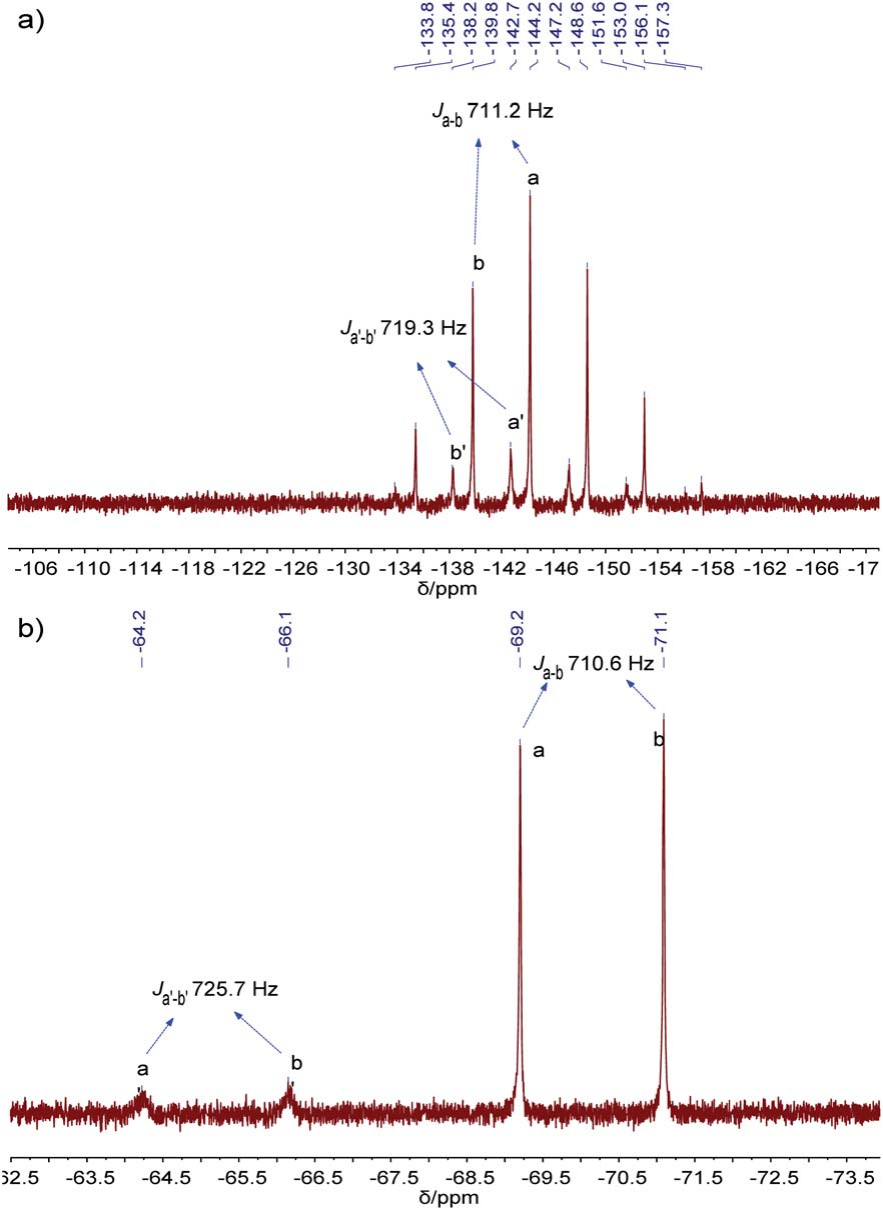
(a) ^31^P NMR (162 MHz, 298 K), and (b) ^19^F NMR (376 MHz, 298 K) spectra of PF_6_^−^@Pd_6_L_12_ in DMSO-*d*_6_.

The resulting cage structure of Pd_6_L_12_ was further investigated to explore its encapsulation of anions. The original nitrate anions are difficult to detect by NMR, so anion exchange of nitrate with other anions were performed. Various anions (20 equiv., excess) were added to a DMSO solution of Pd_6_L_12_ (0.75 mmol L^−1^) and the mixture was allowed to react for 4 h at room temperature, then diethyl ether was added to precipitate out pale yellow solid prior to the acquisition of NMR spectroscopy. After the introduction of NaPF_6_^31^P NMR spectroscopy shows that phosphorus resonances from PF_6_^−^ in DMSO appear expected sharp septet, with the main peak at *δ* −144.19 ppm with coupling constant of *J*_PF_ = 711.2 Hz,^37^ while bound PF_6_^−^ anions display the multiplets centred on *δ* −142.70 ppm, shifted by Δ*δ* = 1.49 ppm with *J*_PF_ = 719.3 Hz to lower field (Fig. 5a and S17†). Moreover, ^19^F NMR shows that one new set of peaks at −65.19 ppm (*J*_FP_ = 725.7 Hz) are downshifted with free PF_6_^−^ (−70.15 ppm, *J*_FP_ = 710.6 Hz) (Fig. 5b and S18†). Additionally for ^1^H NMR H_i_ proton resonance inside the central cavity is upfield shifted by 0.70 ppm (Fig. S19†), while other proton resonances are essentially unaffected. These results demonstrate that two types of PF_6_^−^ anions are present in the solution, some are encapsulated within the cavity, and others are free in the solution.

BF_4_^−^ anions can also be encapsulated inside the cavity. With the addition of KBF_4_ to Pd_6_L_12_, ^19^F NMR shows two signals, one strong signal at −148.27 ppm corresponding well with the peak of free BF_4_^−^ in DMSO-*d*_6_ (−148.3 ppm), and the other new signal at −144.1 ppm (Fig. S20 and S21†). ^11^B NMR show that a new signal at 2.79 ppm is also assigned to bound BF_4_^−^ in the cavity (Fig. S22 and S23†).^38,39^ Simultaneously ^1^H NMR reveals that the proton resonance of H_i_ inside the central cavity is shifted upfield (Δ*δ* = 0.30 ppm) compared to Pd_6_L_12_ (Fig. S19†). These results suggest that some BF_4_^−^ anions are bound within the cavity of Pd_6_L_12_. However, addition of OTf^−^ to Pd_6_L_12_ causes neither new signals nor notable shifts, and ^19^F NMR gives only one sharp signal corresponding to free OTf^−^, suggesting that the nitrate ions in the cage was unaffected by OTf^−^ (Fig. S19 and S24†).

## Conclusions

In summary, we have successfully synthesized Au(i)–NHC bispyridine ligand (L) and Au(i)–NHC-edged Pd_6_L_12_ coordination cage has been assembled from L and Pd^2+^. The cage Pd_6_L_12_ has been fully characterized by NMR, diffusion-ordered NMR spectroscopy, FT-IR, mass spectrometry and cyclic voltammogram. The single crystal X-ray diffraction confirms unequivocally the octahedral structure of Pd_6_L_12_. The octahedral cage has central cavity for guest binding, and has been shown to be capable of encapsulating PF_6_^−^ and BF_4_^−^ anions within the cavity. It paves a way to build NHC-based metal–organic container molecules with large cavity for subsequent guest binding and transformation.

## Experimental

### Synthesis of L

A mixture of c (689.1 mg, 0.715 mmol), 3-pyridylboronic acid (357.5 mg, 2.86 mmol), anhydrous potassium carbonate (988.1 mg, 7.15 mmol), tetrakistriphenylphosphine palladium(0) (0.00715 mmol, 8.3 mg) in 18 mL degassed DMF–H_2_O (v/v = 5 : 1) was stirred at 100 °C for 24 h under argon atmosphere, the progress of the reaction was monitored by TLC. After completion of the reaction, the solvent was removed and the residue was dispersed in water and extracted with CH_2_Cl_2_. The CH_2_Cl_2_ solution was washed with water and brine, dried over anhydrous MgSO_4_, filtered and concentrated *in vacuo*. Purification by column chromatography on silica gel (ethyl acetate : hexane 1 : 1) to give the product as white powder which was further purified by washing with hexane (464 mg, 75%). ^1^H NMR (400 MHz, DMSO-*d*_6_): *δ* 9.05 (s, 2H), 8.64 (s, 2H), 8.27–8.22 (m, 2H), 8.08 (s, 2H), 7.76 (s, 4H), 7.57–7.53 (m, 2H), 2.59 (dt, *J* = 14.0, 6.9 Hz, 4H), 1.36 (d, *J* = 6.8 Hz, 12H), 1.30 (d, *J* = 6.9 Hz, 12H). ^13^C NMR (101 MHz, CDCl_3_): *δ* 185.55, 149.02, 148.43, 146.49, 140.41, 136.25, 134.79, 133.79, 123.65, 123.29, 123.14, 29.04, 24.48, 24.06. Q-TOF-MS(ESI^+^) (*m*/*z*): calcd. for [M + H]^+^ [C_37_H_43_N_4_AuI + H]^+^ 867.2198, found 867.2206 and calcd for [M–AuI + H]^+^ [C_41_H_44_N_2_O_2_ + H]^+^ 543.3488, found 543.3471.

### Synthesis of Pd_6_L_12_

L (174.0 mg, 0.2 mmol) and Pd(NO_3_)_2_·2H_2_O (27.0 mg, 0.1 mmol) were dissolved in DMSO (10.0 mL) were mixed. Each time 1 mL of the mixture was taken and stirred at 70 °C for 6 h. The quantitative formation of Pd_6_L_12_ was confirmed by ^1^H NMR and ^1^H DOSY NMR. The addition of diethyl ether to the yellow homogeneous solution precipitated the product, and it was collected by filtration, washed with diethyl ether, and dried *in vacuo* to give a pale yellow solid (13.8 mg, 70%). ^1^H NMR (500 MHz, DMSO-*d*_6_): *δ* 9.74 (s, 2H), 9.08 (s, 2H), 8.60 (s, 2H), 7.88 (d, *J* = 39.1 Hz, 8H), 2.55 (s, 4H), 1.35 (s, 24H). ^1^H DOSY NMR (400 MHz, DMSO-*d*_*6*_): *D* = 7.16 × 10^−11^ m^2^ s^−1^. Pd_6_L_12_ (PF_6_^−^ salt) was prepared by adding excess NaPF_6_ to the DMSO solution of Pd_6_L_12_. Electrospray ionization mass spectra were recorded for Pd_6_L_12_ (PF_6_^−^ salt). *m*/*z* calcd for C_444_H_504_N_48_Au_12_I_12_P_8_F_48_Pd_6_ [M–4(PF_6_)]^4+^ 3049.4240, found 3049.4123; *m*/*z* calcd for C_444_H_504_N_48_Au_12_I_12_P_7_F_42_Pd_6_ [M–5(PF_6_)]^5+^ 2410.5462, found 2410.5425; *m*/*z* calcd for C_444_H_504_N_48_Au_12_I_12_P_6_F_36_Pd_6_ [M–6(PF_6_)]^6+^ 1984.6278, found 1984.6254; *m*/*z* calcd for C_444_H_504_N_48_Au_12_I_12_P_5_F_30_Pd_6_ [M–7(PF_6_)]^7+^ 1680.4003, found 1680.3982; *m*/*z* calcd for C_444_H_504_N_48_Au_12_I_12_P_4_F_24_Pd_6_ [M–8(PF_6_)]^8+^ 1452.2296, found 1452.2276; *m*/*z* calcd for C_74_H_84_N_8_Au_2_I [2L–I]^+^ 1605.5190, found 1605.5175.

### X-ray crystallography for Pd_6_L_12_

Quality single crystals were obtained by the slow diffusion of ethyl acetate vapour into a DMSO solution of Pd_6_L_12_ (NO_3_^−^ salt) to give colourless crystals of Pd_6_L_12_ over one month. A crystal was picked (0.2 × 0.2 × 0.2 mm^3^) and coated in paratone oil, attached to a glass silk which was inserted in a stainless steel stick, then quickly transferred to the Agilent Technologies SuperNova X-ray diffractometer with the Enhance X-ray Source of Cu Kα radiation (*λ* = 1.54184 Å) using the *ω*–*ϕ* scan technique. Data collection were measured at 150.00 (10) K. The unit cell parameters were solved by direct methods and the unit cell parameters refined against all data by anisotropic full-matrix least-squares methods on *F*^2^ with the SHELXL program.^40^ Hydrogen atoms were calculated in ideal positions (riding model). All nitrate ions and solvent molecules could not be reasonably located because of highly disordered structures, and were removed by PLATON/SQUEEZE routine.^41^

Crystallographic data for Pd_6_L_12_: C_74_H_84_Au_2_I_2_N_8_Pd FW = 1839.62, trigonal, *R*3̄, *a* = 29.8768 (16) Å, *b* = 29.8768 (16) Å, *c* = 74.341 (3) Å, *α* = 90°, *β* = 90°, *γ* = 120°, *V* = 57 468 (7) Å^3^, *Z* = 18, *T* = 150.00 (10) K, *λ* = 1.54184 Å, *ρ*_calcd_ = 0.957 mg m^−3^, *μ* = 9.349 mm^−1^, 13 205 reflections were collected (7693 were unique) for 6.856 < 2*θ* < 79.938, *R*(int) = 0.0372, *R*_1_ = 0.0888, w*R*_2_ = 0.2668 [*I* ≥ 2*σ*(*I*)], *R*_1_ = 0.1033, w*R*_2_ = 0.2821 (all data) for 725 parameters and 825 restraints, GOF = 1.059. Selected bond lengths and bond angles are presented in Table S1.† The crystal structure was submitted to the Cambridge Structural Database under the CCDC number 2013514.

## Conflicts of interest

There are no conflicts to declare.

## Supplementary Material

RA-010-D0RA07509D-s001

RA-010-D0RA07509D-s002
