# Supplementary material for: Confinement of a Au–N-heterocyclic carbene in a Pd6L12 metal–organic cage
Source: RSC Adv. 2020 Oct 27;10(64):39323–7. doi: 10.1039/d0ra07509d (PMC9057365; doi:10.1039/d0ra07509d)
Supplement: RA-010-D0RA07509D-s001 [file RA-010-D0RA07509D-s001.pdf]

Electronic Supplementary Material (ESI) for RSC Advances.
This journal is © The Royal Society of Chemistry 2020

Electronic Supplementary Information

Confinement of Au-*N*-heterocyclic carbene in Pd_6L_{12} metal-organic cage

Lihua Zeng, ‡ Shujian Sun,‡ Zhang-Wen Wei, Yu Xin, Liping Liu and Jianyong Zhang*

MOE Laboratory of Polymeric Composite and Functional Materials, School of Materials Science and Engineering, School of Chemistry, Sun Yat-Sen University, Guangzhou 510275, China. E-mail: zhjyong@mail.sysu.edu.cn.

Additional experimental section

General methods

All commercial chemicals and solvents were purchased without further purification, unless otherwise specified. ^{1}H NMR, ^{11}B NMR, ^{19}F NMR, ^{31}P NMR,^{1}H-^{1}H COSY and ^{1}H-^{1}H NOESY spectra were recorded on a Bruker Avance III 400MHz NMR spectrometer. ^{1}H DOSY spectrum was recorded on a Bruker Avance III 600MHz NMR spectrometer. ESI-TOF mass spectra were performed on a timsTOF from Bruker equipped with an auto-sample injection system. Detection was in positive-ion mode and the capillary voltage was 3500 V, dry temperature was held at 200 ºC, dry gas was 4.5 L min^{-1}, and Nebulizer was 0.6 bar. For cyclic voltammetry the working electrode was glassy carbon electrode 3 mm in diameter, a platinum wire was counter electrode, and an Ag/AgCl (3.0 mol L^{-1} KCl) electrode or Ag wire as reference electrode. All electrochemical experiments were carried out in dry DMSO solutions of n-Bu_4NPF_6 (0.10 mol L^{-1}) as supporting electrolyte, which was freed of oxygen by bubbling through high-purity nitrogen before experiments. The concentration was 3.0 μmol L^{-1} for **L** and 0.25 μmol L^{-1} for Pd$_6$**L**$_{12}$.

Synthesis of 4-iodo-2,6-diisopropylaniline

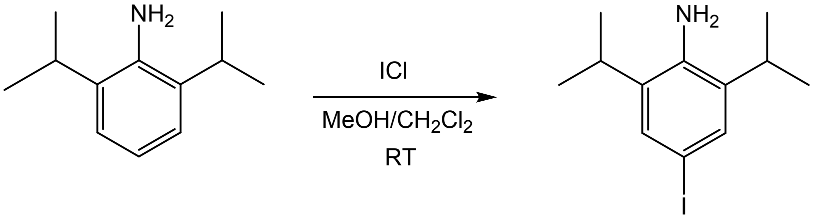

Synthesized according to the reported procedure.[S1] 2,6-Diisopropylaniline (9.399 g, 53.1 mmol) was dissolved in a mixture of methanol and dichloromethane (60 mL, *v/v* 1:1). Iodochloride (9.464 g, 58.4 mmol, excess) dissolved in a mixture of methanol-dichloromethane (60 mL, *v/v* 1:1) was added into the solution of 2,6-diisopropylaniline. The resultant mixture was stirred at room temperature for 12 h. At the end of the reaction the solvent was evaporated out. The resulting crude solid product was treated with 60 mL saturated solution of $Na_2S_2O_3$. The resulting mixture was stirred at room temperature for 0.5 h and then extracted with dichloromethane (3 × 20 mL). After drying over sodium sulfate, the solvent was evaporated out. Brown oil was eventually obtained after distillation under reduced pressure (15.149 g, 94%). ^{1}H NMR (400 MHz, $CDCl_3$) δ 7.33 (s, 2H), 3.73 (s, 2H), 2.89 (m, 2H), 1.29 (d, J = 6.9 Hz, 12H).

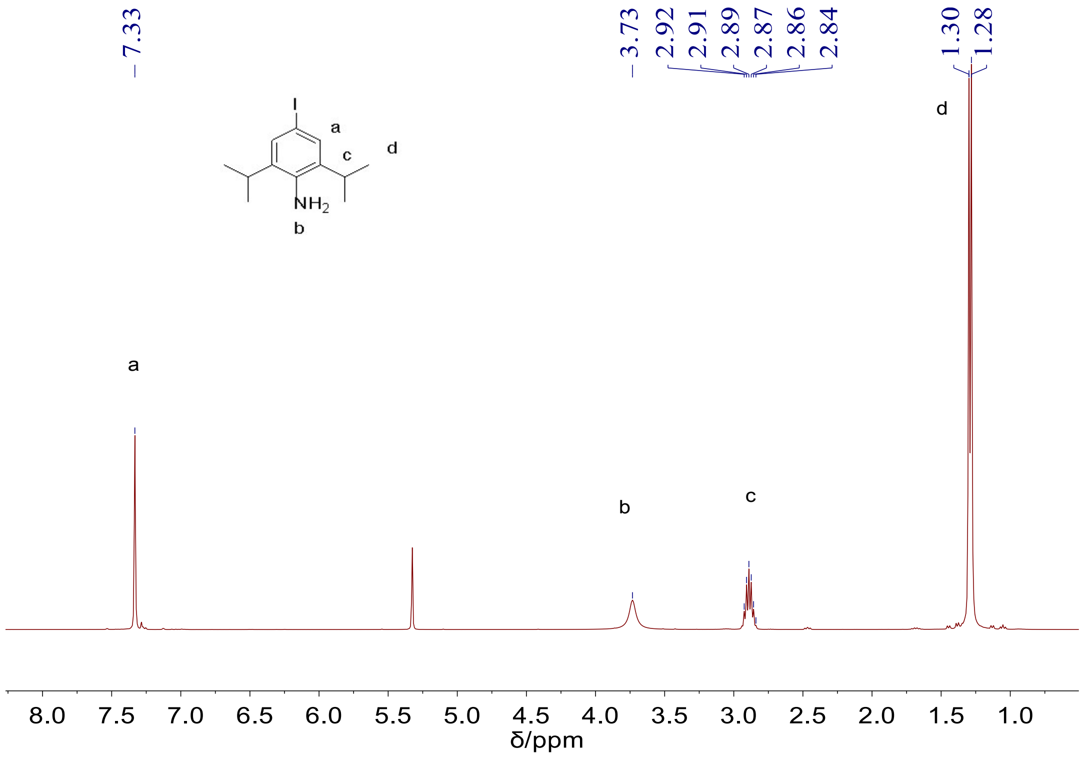

Fig. S1 ^{1}H NMR spectrum of 4-iodo-2,6-diisopropylaniline (400 MHz, $CDCl_3$).

Synthesis of bis(4-iodo-2,6-diisopropylphenyl)diazabutadiene (a)

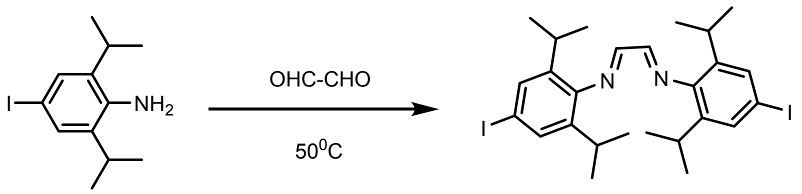

a

Synthesized according to the reported procedure.[S1] Glyoxal (5.257 g, 40 wt% in water, 36.3 mmol) was added dropwise into 4-iodo-2,6-diisopropylaniline (21.969 g, 72.5 mmol) in methanol (36 mL), 362 μL acetic acid was added in the system as catalyst. The reaction mixture was stirred for 7 h at 50 °C. The yellow solid was achieved by filtration and washed with methanol (3 × 10 mL), leading to the yellow product (13.939 g, 61%). ^{1}H NMR (400 MHz, $CDCl_3$): δ 8.06 (s, 2H), 7.49 (s, 4H), 2.86 (m, 4H), 1.20 (d, J = 6.9 Hz, 24H).

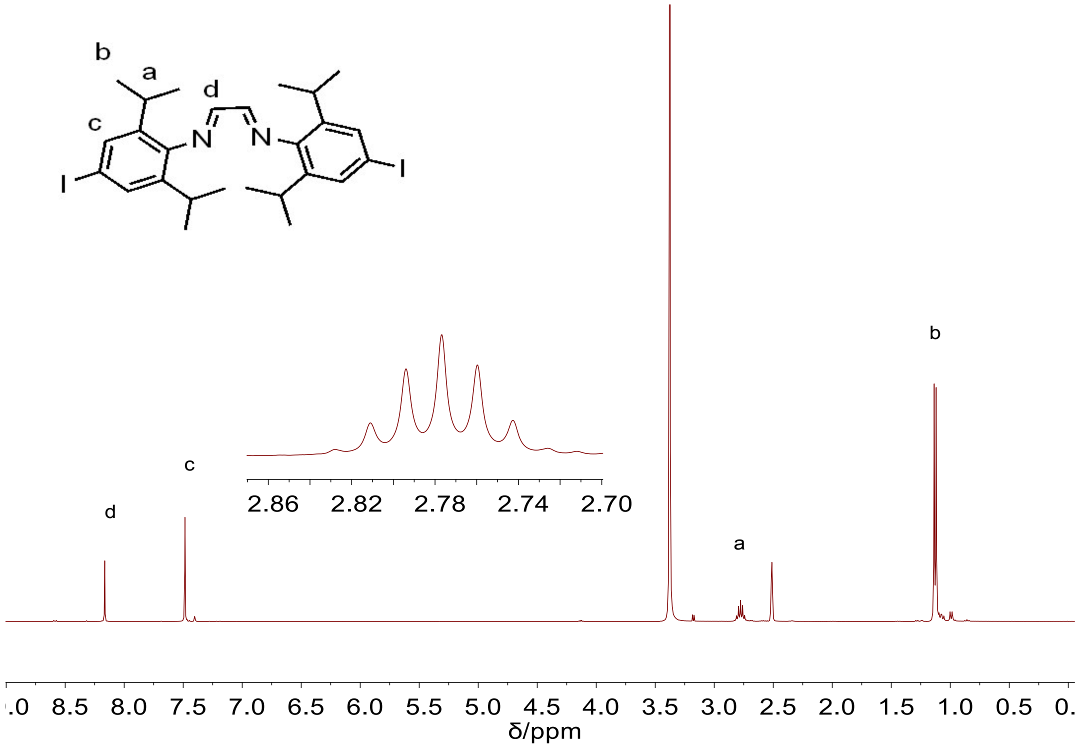

Fig. S2 ^{1}H NMR spectrum of bis (4-iodo-2,6-diisopropylphenyl)diazabutadiene (400 MHz, DMSO-d_6).

Synthesis of *N*,*N*'-1,3-bis(4-iodo-2,6-diisopropylphenyl)imidazolium chloride (b)

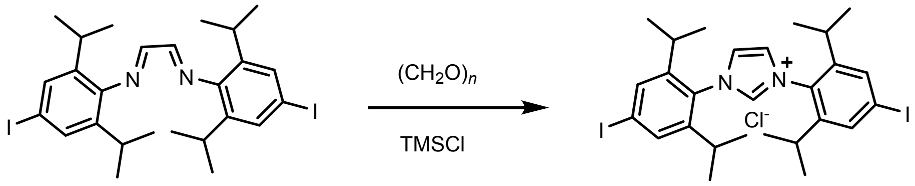

a **b**

A 250 mL round-bottom flask equipped with a magnetic stirring bar and a stopper was charged with ethyl acetate (100 mL) and heated in an oil bath at 70 °C. To the warm solvent bis(4-iodo-2,6-diisopropylphenyl)diazabutadiene (6.910 g, 11 mmol) and paraformaldehyde (0.360 g, 12 mmol, excess) were added. Next, a solution of chlorotrimethylsilane (1.5 mL) in ethyl acetate (2.5 mL) was added dropwise over 45 min with vigorous stirring. The resulting yellow-orange suspension was further stirred for 4 h at 70 °C. The color turned to black-brown. The suspension was filtered through a funnel and the white precipitate was rinsed twice with ethyl acetate (3 × 10 mL) to obtain the product (7.264 g, 62%). ^{1}H NMR (400 MHz, DMSO-d_6): δ 10.34 (s, 1H), 8.58 (s, 2H), 7.87 (s, 4H), 2.26 (m, 4H), 1.24 (d, J = 6.8 Hz, 12H), 1.14 (d, J = 6.8 Hz, 12H). ^{13}C NMR (100 MHz, DMSO-d_6): δ 147.54 (s), 139.60 (s), 133.99 (s), 130.24 (s), 126.60 (s), 99.99 (s), 29.05

(s), 24.24 (s), 23.22 (s). FT-IR (cm^{-1}, KBr): 1623 (w) (C=N), 1568 (m), 1532 (m), 1464 (m), 1328 (m), 1205 (s), 1107 (w), 949 (s), 862 (s), 787 (w), 769 (w), 686 (w), 565 (w). TOF-MS (ESI+): *m*/*z* found (calcd.) for $[M-Cl]^+$ $[C_{27}H_{35}N_2I_2]^+$ = 641.0879 (641.0884).

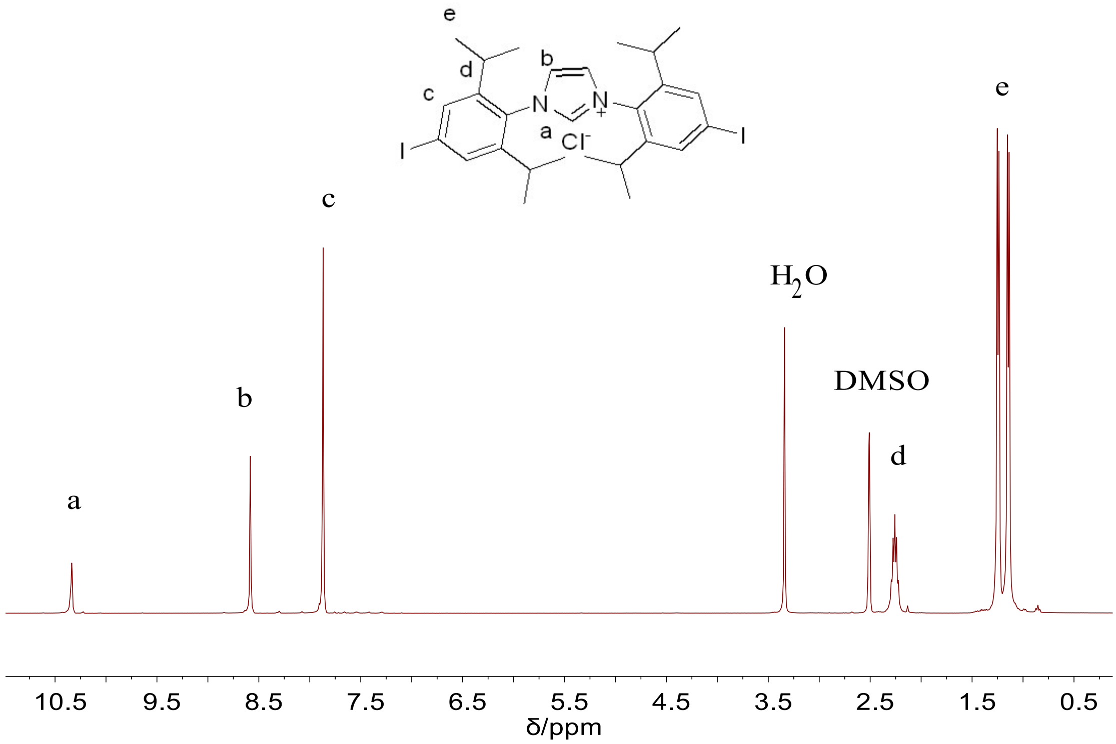

Fig. S3 ^{1}H NMR spectrum of 1,3-bis(4-iodo-2,6-diisopropylphenyl)imidazolium chloride (400 MHz, DMSO-d_6).

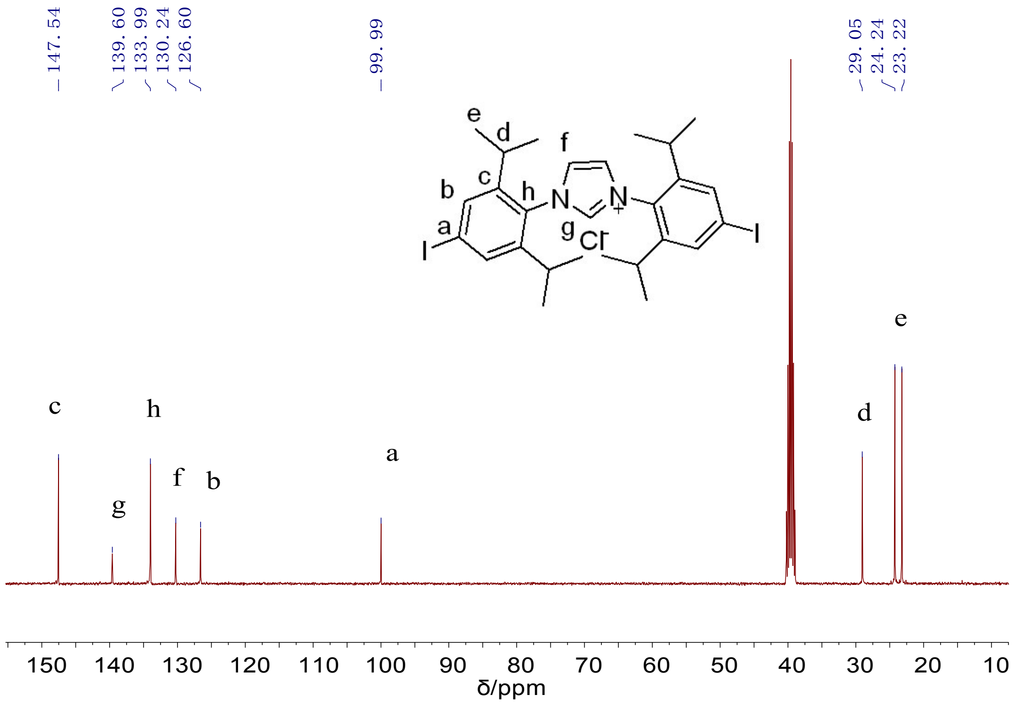

Fig. S4 ^{13}C NMR spectrum of 1,3-bis(4-iodo-2,6-diisopropylphenyl)imidazolium chloride (100 MHz, DMSO-d_6).

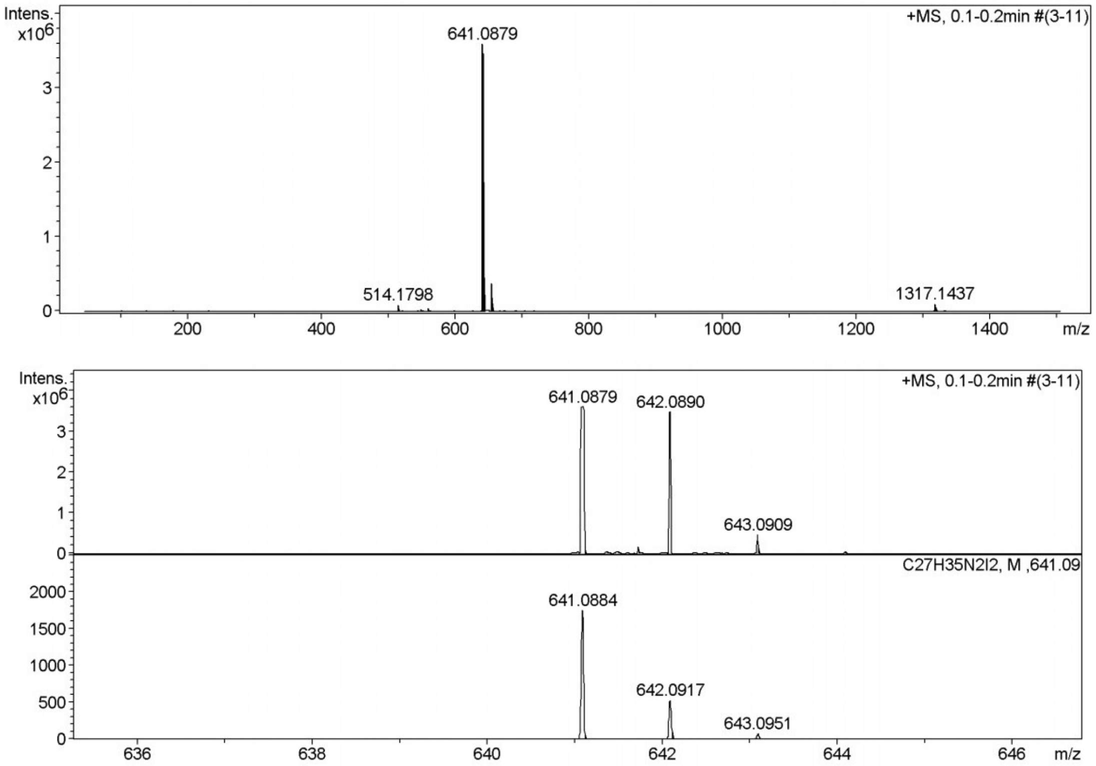

Fig. S5 ESI-MS spectrum of1,3-bis(4-iodo-2,6-diisopropylphenyl)imidazolium chloride in CH_3CN, and isotopic distribution and simulation of $[M-Cl]^+$ $[C_{27}H_{35}N_2I_2]^+$.

Synthesis of (1,3-bis(4-iodo-2,6-diisopropylphenyl)-1H-imidazol-2(3H)-ylidene)gold(I) chloride (c)

Synthesized according to modified literature method.[S2] Compound **b** (2.57 mmol, 1.737 g), Na_2CO_3 (25.7 mmol, 2.724 g) and $HAuCl_4·4H_2O$ (2.57 mmol, 1.058 g) were added to a dry Schlenk vial equipped with a magnetic stirring bar under argon atmosphere. Then 3-chloropyridine (12.26 g, 108 mmol) was added dropwise, and the reaction mixture was stirred at 80 °C for 24 h. After completion of reaction, the solution was cooled to room temperature and 250 mL dichloromethane was added to dissolve the product, then the mixture was filtered through celite. The combined organic phase was evaporated to dryness under reduced pressure. The residue was washed with MeOH and dried under vacuum to give **c** as light yellow solid (1.424 g, 63%). ^{1}H NMR (400 MHz, $CDCl_3$): δ 7.62 (s, 4H), 7.17 (s, 2H), 2.47 (p, J = 6.8 Hz, 4H), 1.34 (d, J = 6.8 Hz, 12H), 1.22 (d, J = 6.8 Hz, 12H). TOF-MS (ESI+): m/z found (calcd.) for $[M+Na]^+$ $[C_{27}H_{34}AuClN_2I_2Na]^+$ = 895.0048 (895.0058).

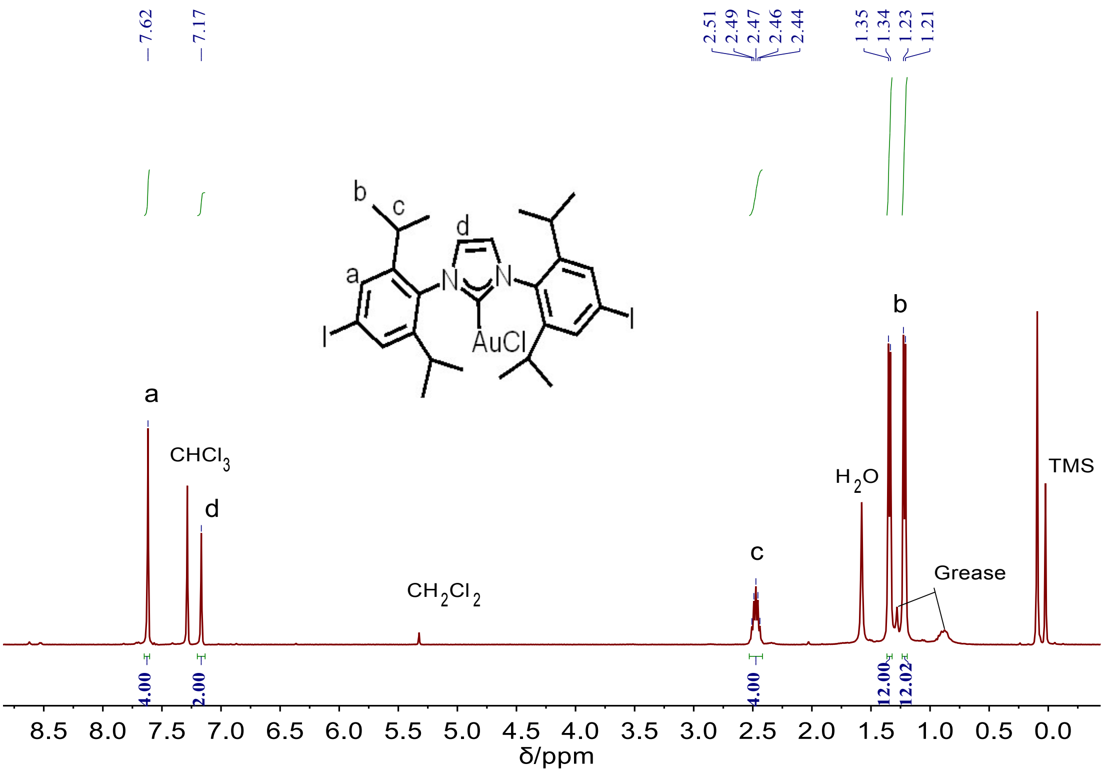

Fig. S6 ^{1}H NMR spectrum of **c** in $CDCl_3$ (400 MHz, 298 K).

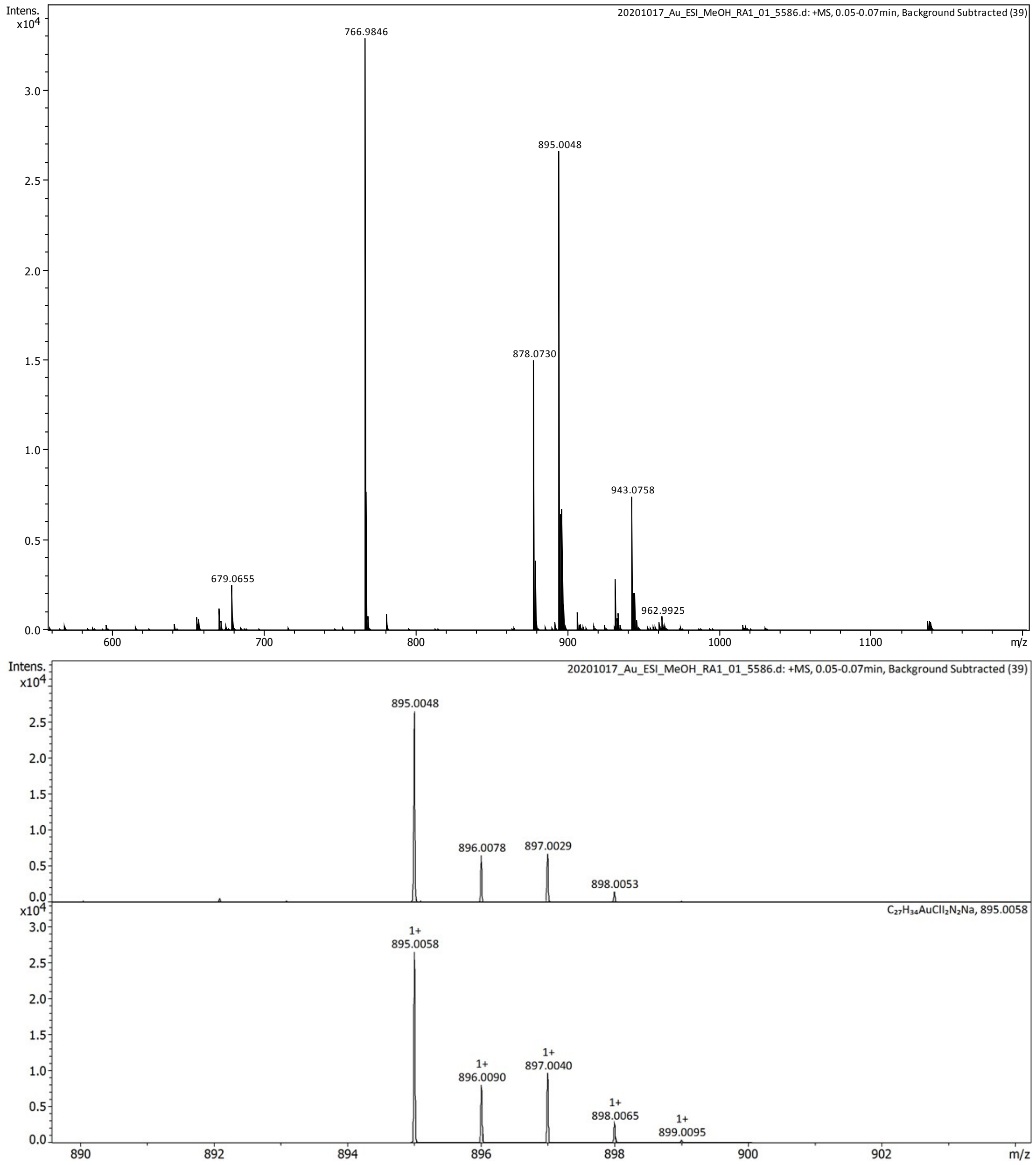

Fig. S7 ESI-MS spectrum of **c** in CH_3OH, and isotopic distribution and simulation of $[M+Na]^+$ $[C_{27}H_{34}AuClN_2I_2Na]^+$.

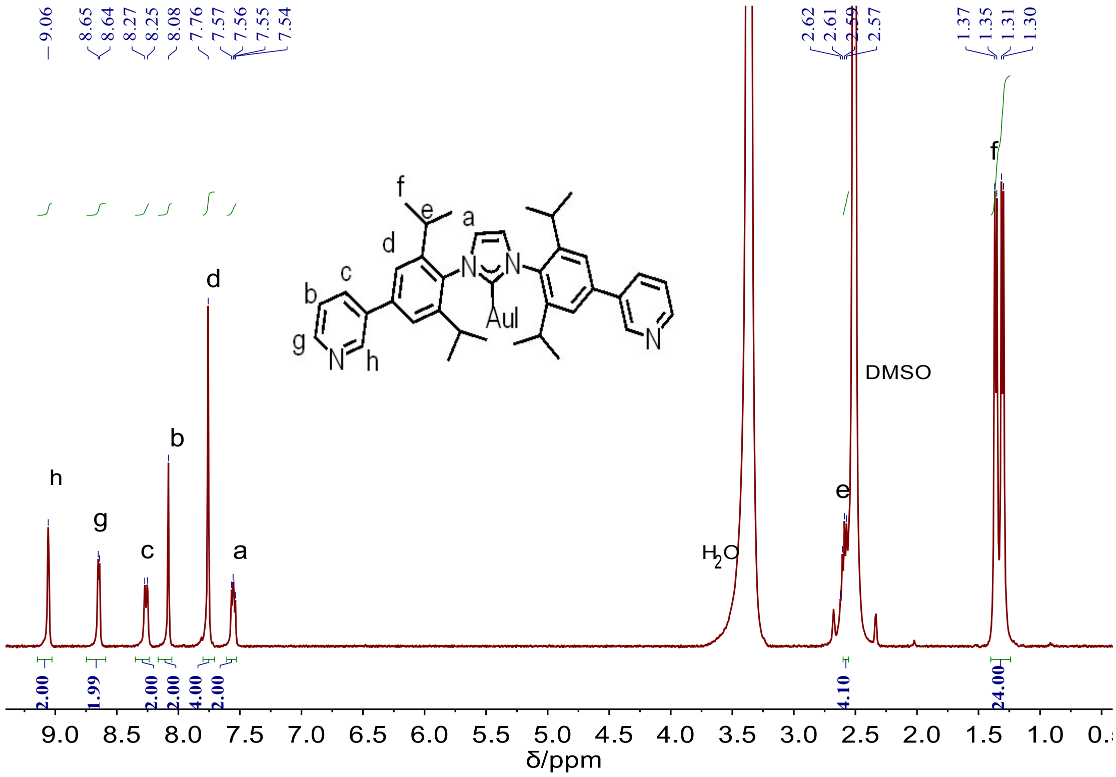

Fig. S8 ^{1}H NMR spectrum of **L** in DMSO-d_6 (400 MHz, 293 K).

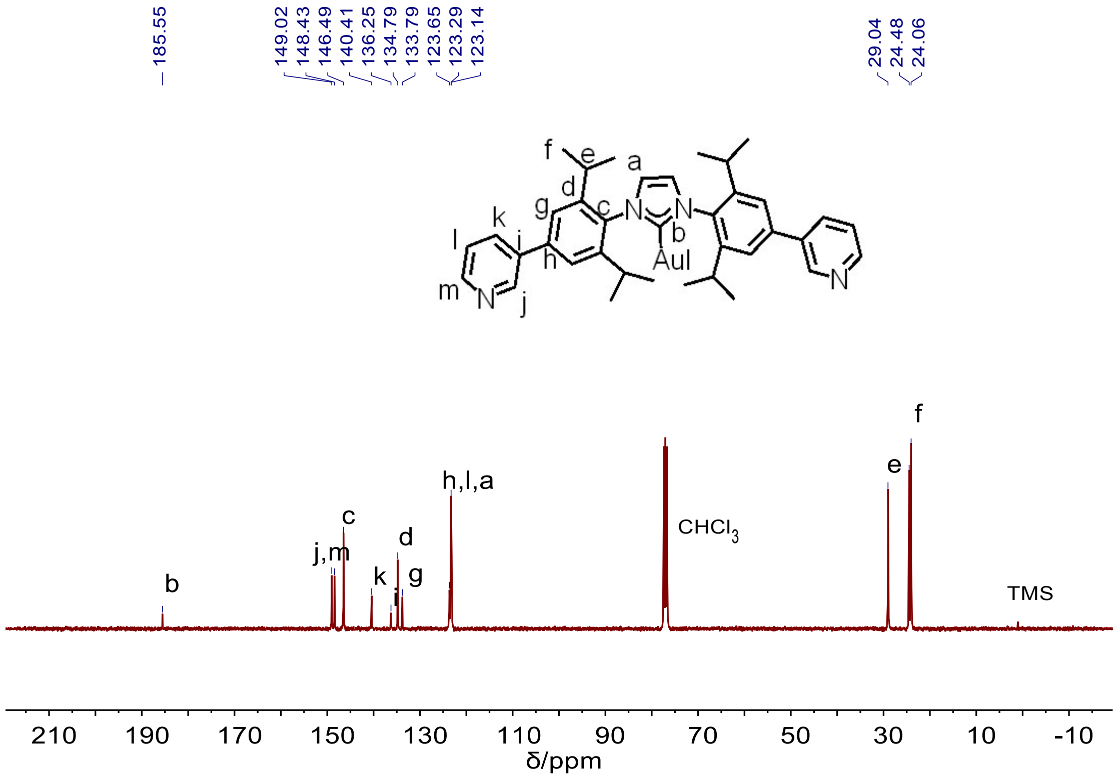

Fig. S9 ^{13}C NMR spectrum of **L** in $CDCl_3$ (100 MHz, 298 K).

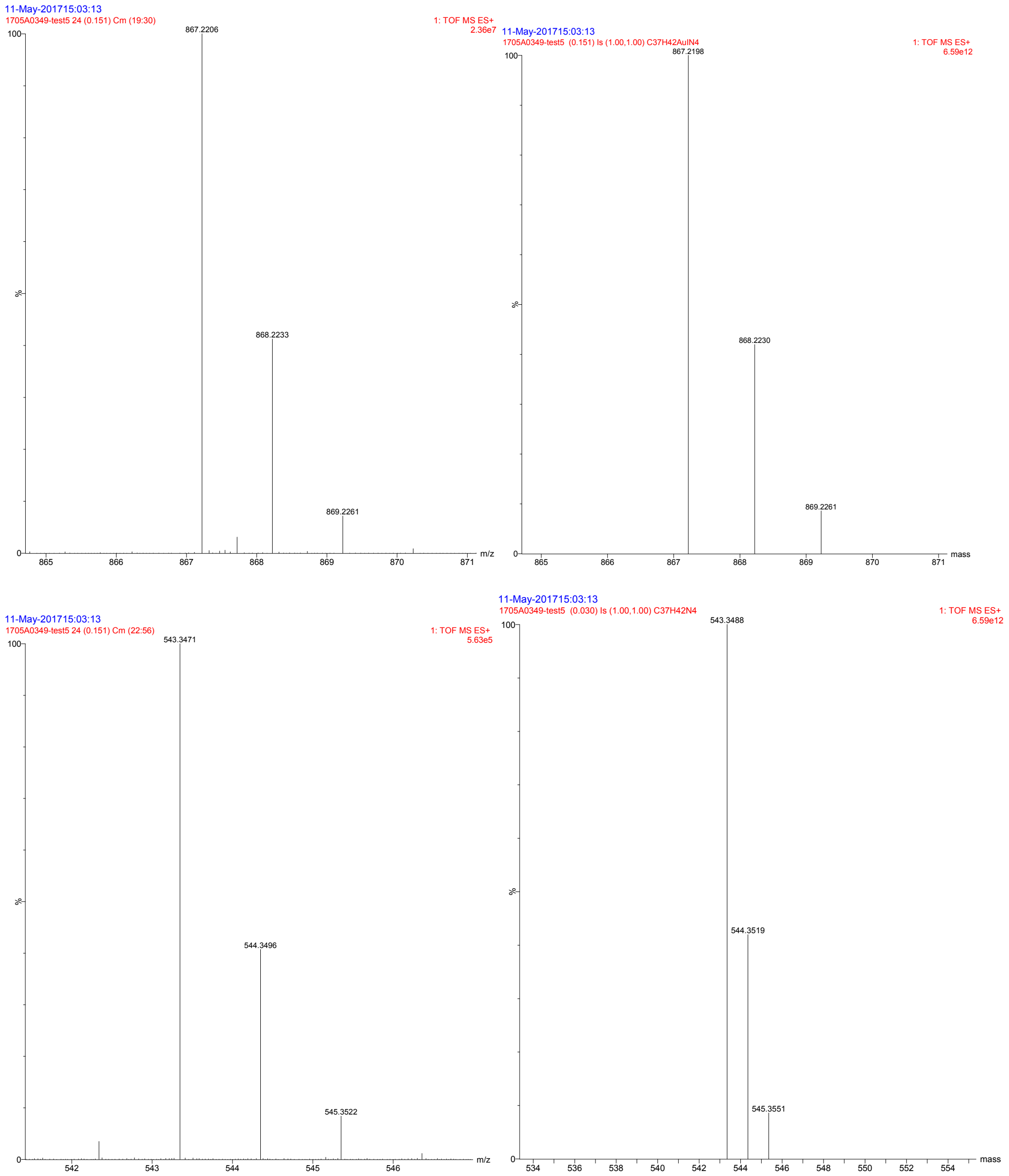

Fig. S10 Q-TOF-MS spectrum of **L** in MeCN, and isotopic distribution and simulation of $[M+H]^+$ $[C_{37}H_{42}N_4AuI+H]^+$ found (calcd.) for 867.2206 (867.2198) and $[M–AuI+H]^+$ $[C_{41}H_{44}N_2O_2+H]^+$ found (calcd.) for 543.3471 (543.3488).

References

S1 H. Yang, G. Li, Z. Ma, J. Chao and Z. Guo, *J. Catal.*, 2010, **276**, 123-133.

S2 W. Wang, A. Zheng, P. Zhao, C. Xia and F. Li, *ACS Catal.*, 2014, **4**, 321-327.

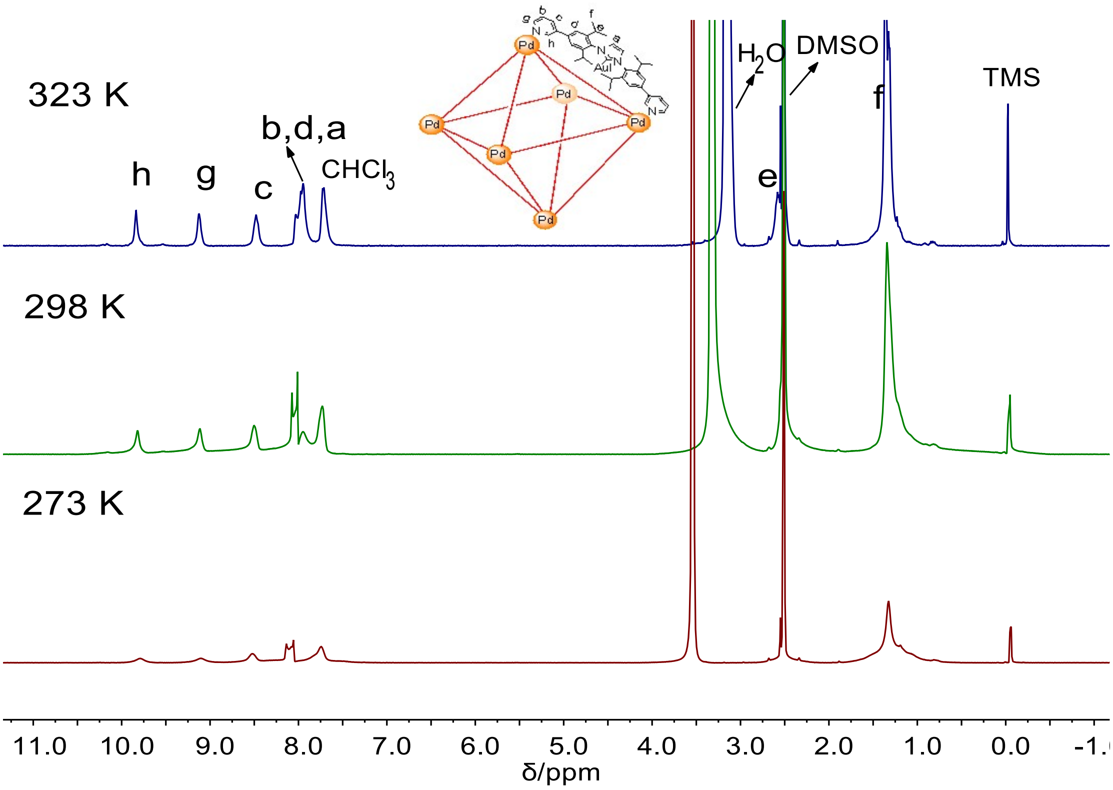

Fig. S11 VT ^{1}H NMR spectra of $Pd_6\mathbf{L}_{12}$ in $CDCl_3$-DMSO-d_6 solvent mixture (*v*/*v* 1:1) (400 MHz).

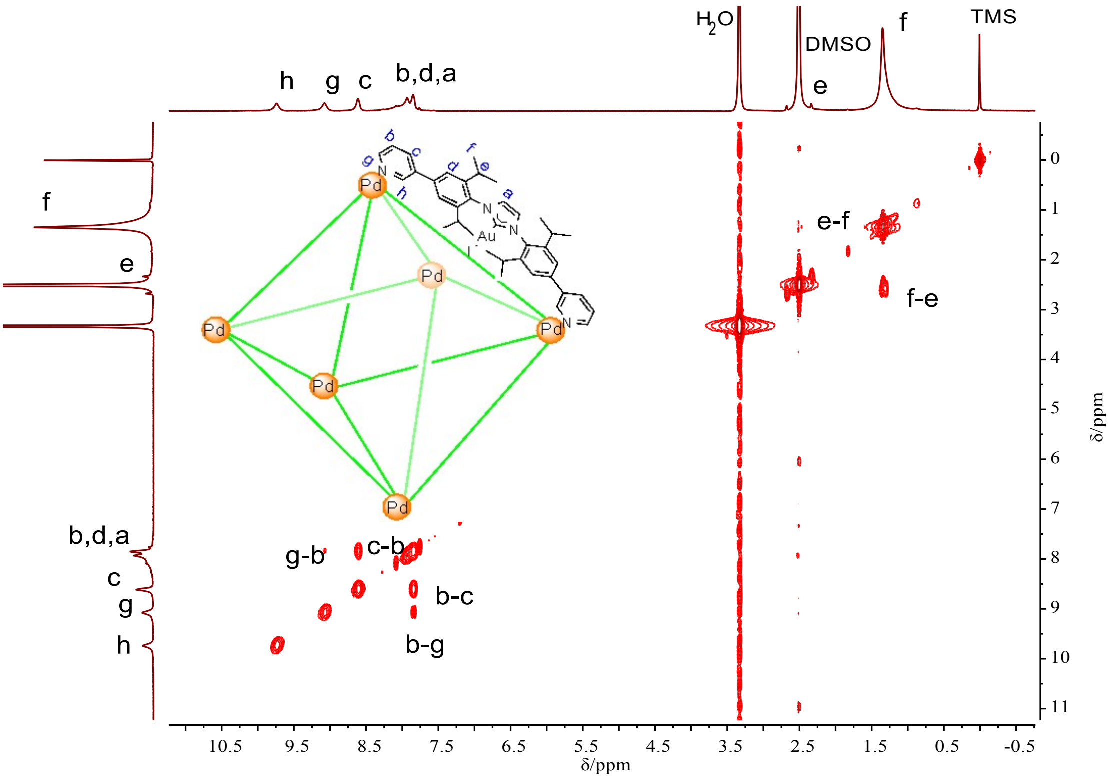

Fig. S12 ^{1}H-^{1}H COSY spectrum of $Pd_6\mathbf{L}_{12}$ in DMSO-d_6 (400 MHz, 298 K).

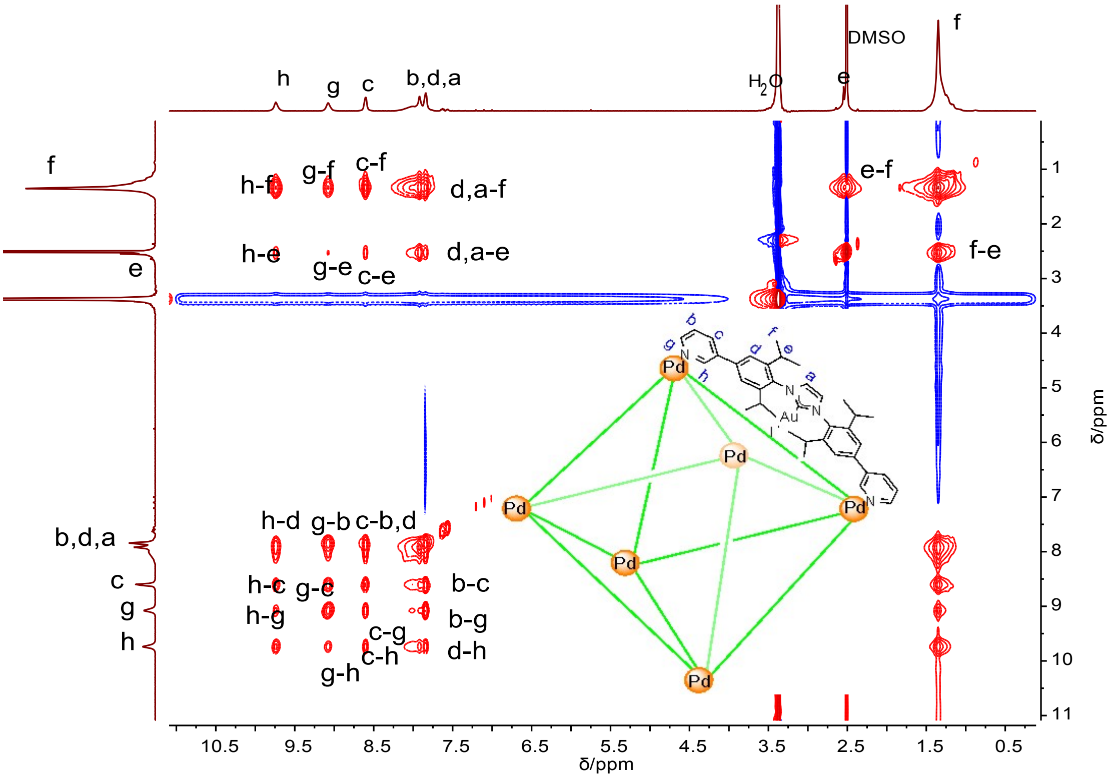

Fig. S13 ^{1}H-^{1}H NOESY spectrum of Pd$_6$**L**$_{12}$ in DMSO-d_6 (500 MHz, 298 K).

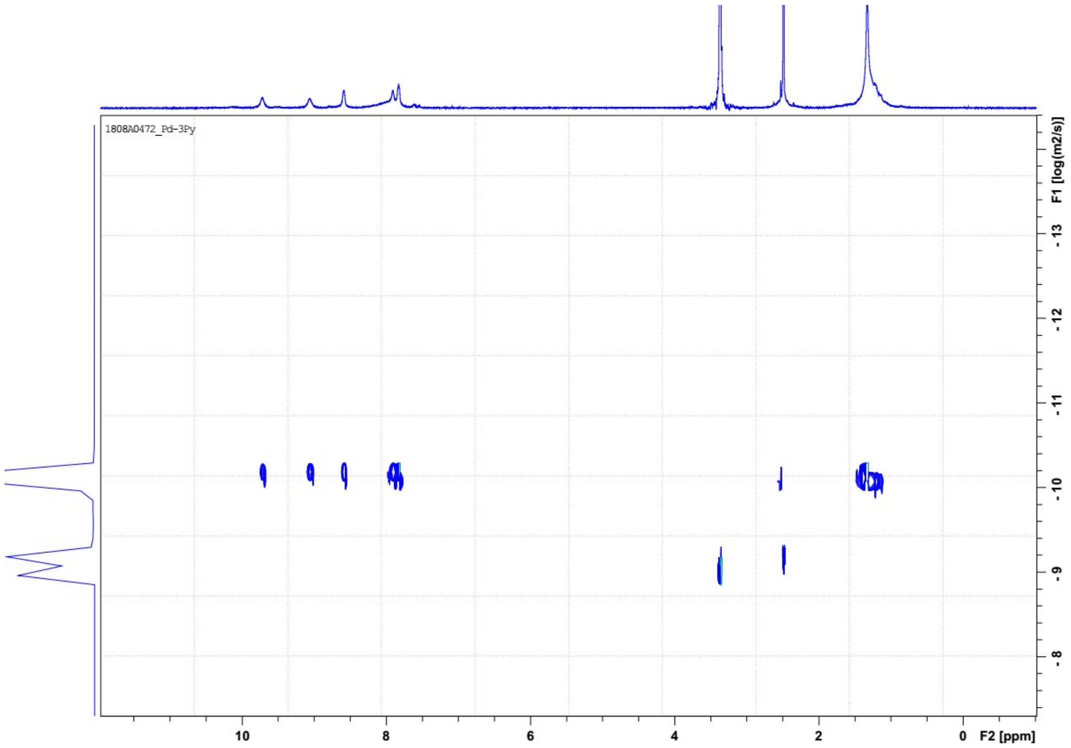

Fig. S14 ^{1}H DOSY spectrum of Pd$_6$**L**$_{12}$ in DMSO-d_6 (500 MHz, 298 K). The diffusion coefficient is 5.75×10^{-11} m^2 s^{-1}, the dynamic diameter is 19.0 Å. Stokes-Einstein equation: $D = \frac{k_b T}{6\pi\eta r}$ was applied to estimate the dynamic radius, with D is diffusion values (5.75×10^{-11} m^2 s^{-1}) obtained from DOSY spectrum, k_b is Boltzmann constant (1.38×10^{-23} N m K^{-1}), T is absolute temperature

(298 K), η is dynamic viscosity of DMSO-d_6, 1.996 mPa s^{-1}, and r is the estimated dynamic radius. According to the Stokes-Einstein equation, it could be calculated that the molecular radius of $Pd_6\mathbf{L}_{12}$ was 19.0 Å.

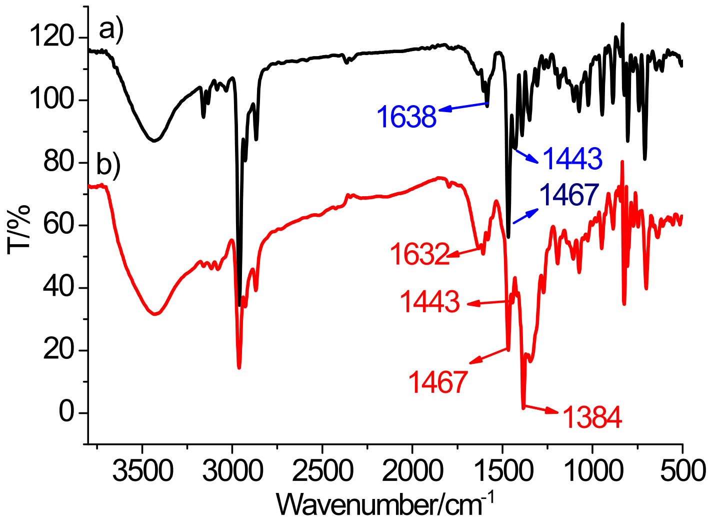

Fig. S15 FT-IR spectra of a) **L** and b) $Pd_6\mathbf{L}_{12}$.

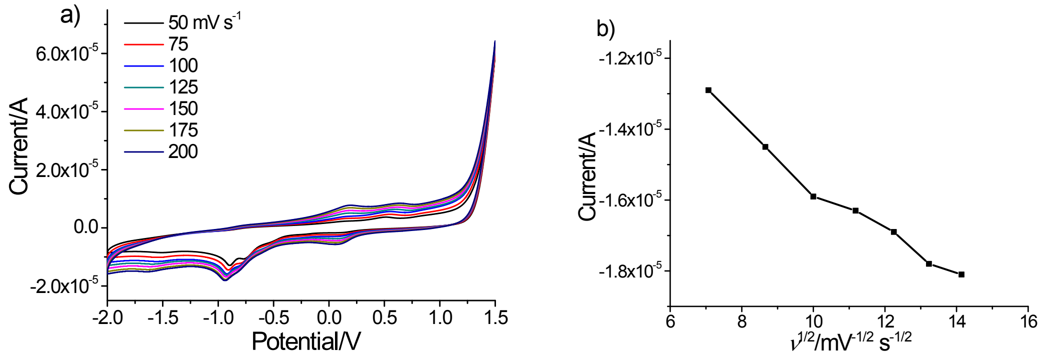

Fig. S16 (a) Cyclic voltammograms of 0.25 μmol L^{-1} $Pd_6\mathbf{L}_{12}$ in DMSO at scan rates of 50, 75, 100, 125, 150, 175 and 200 mV s^{-1}, and (b) plot of the reduction current versus the square root of the scan rate ($v^{1/2}$).

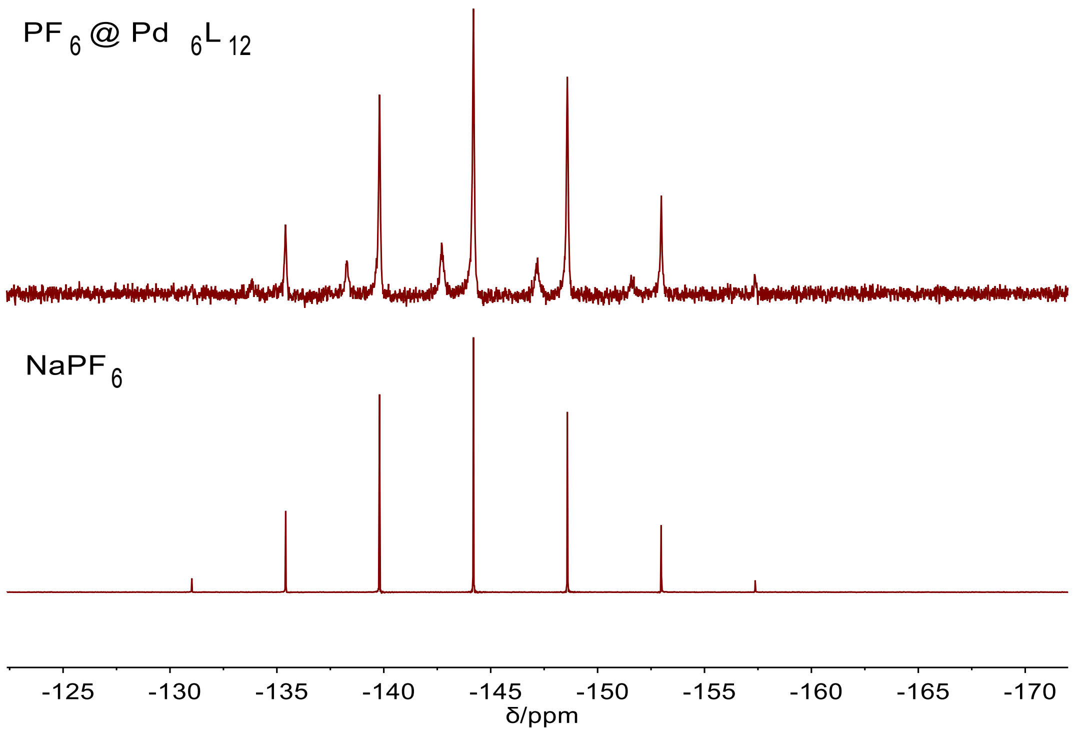

Fig. S17 ^{31}P NMR spectra of PF_6^-@Pd_6**L**$_{12}$ (top) and $NaPF_6$ (down) in DMSO-d_6 (162 MHz, 298 K).

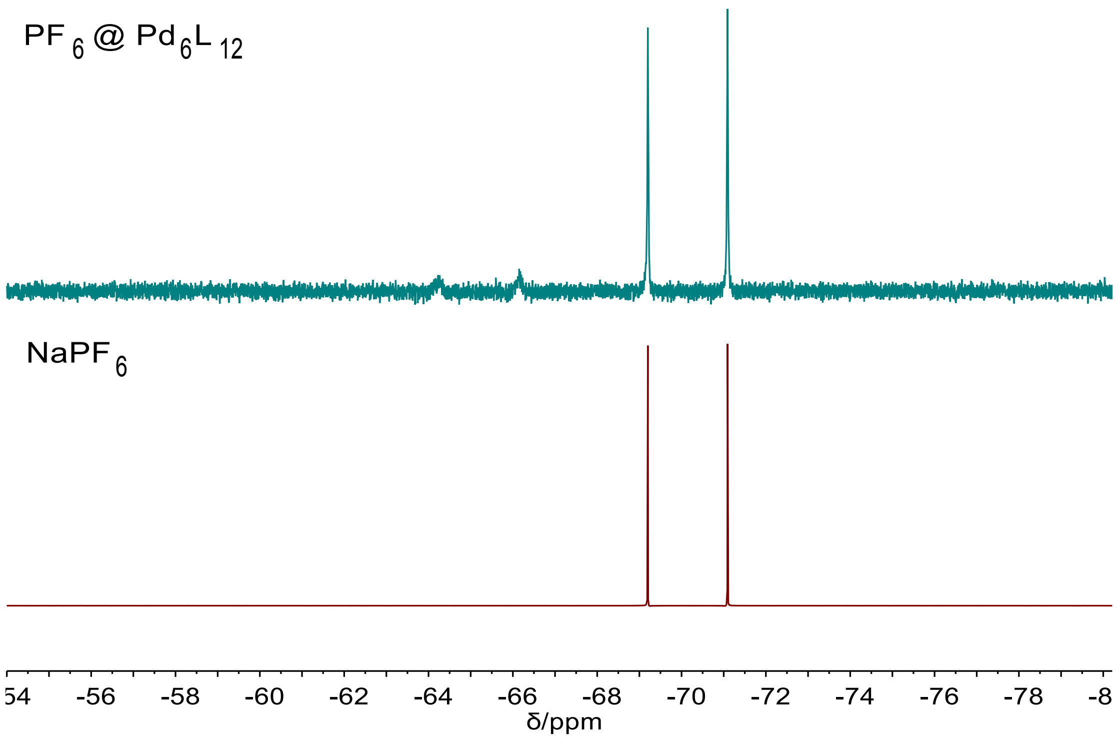

Fig. S18 ^{19}F NMR spectra of PF_6^-@Pd_6**L**$_{12}$ (top) and $NaPF_6$ (down) in DMSO-d_6 (376 MHz, 298 K).

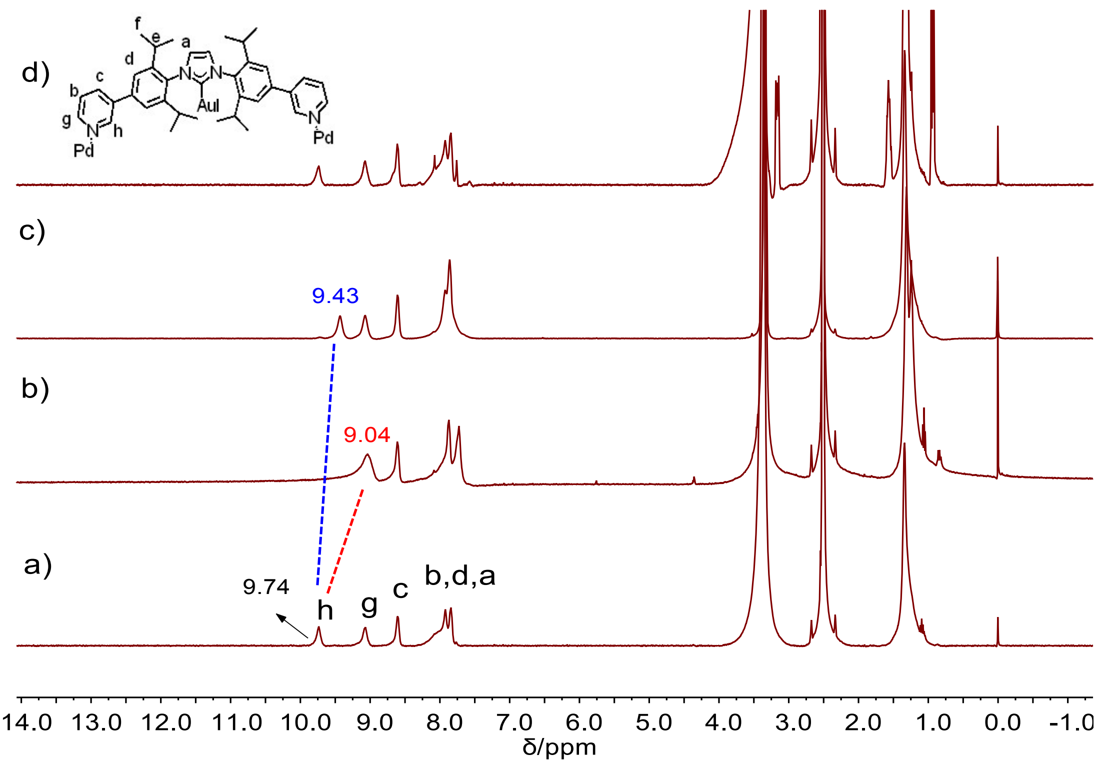

Fig. S19 ^{1}H NMR spectra of (a) $Pd_6\mathbf{L}_{12}$, (b) $PF_6^-@Pd_6\mathbf{L}_{12}$, (c) $BF_4^-@Pd_6\mathbf{L}_{12}$, (d) $OTf^-@Pd_6\mathbf{L}_{12}$ in DMSO-d_6 (400 MHz, 298 K).

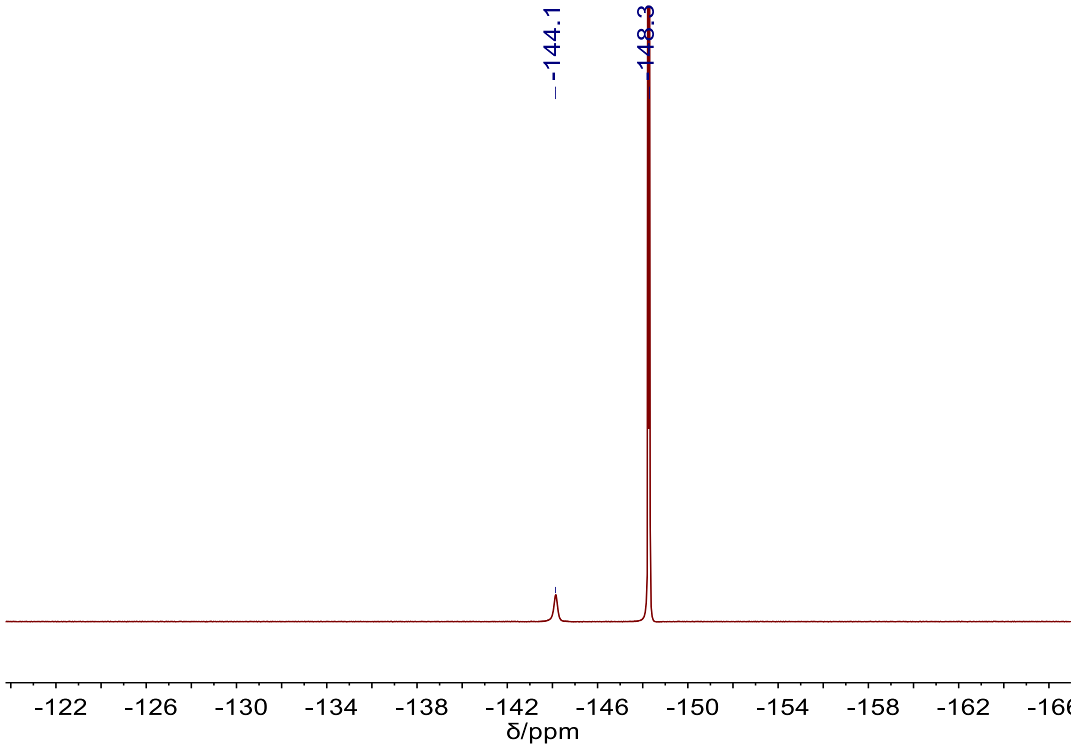

Fig. S20 ^{19}F NMR spectrum of $BF_4^-@Pd_6\mathbf{L}_{12}$ in DMSO-d_6 (376 MHz, 298 K).

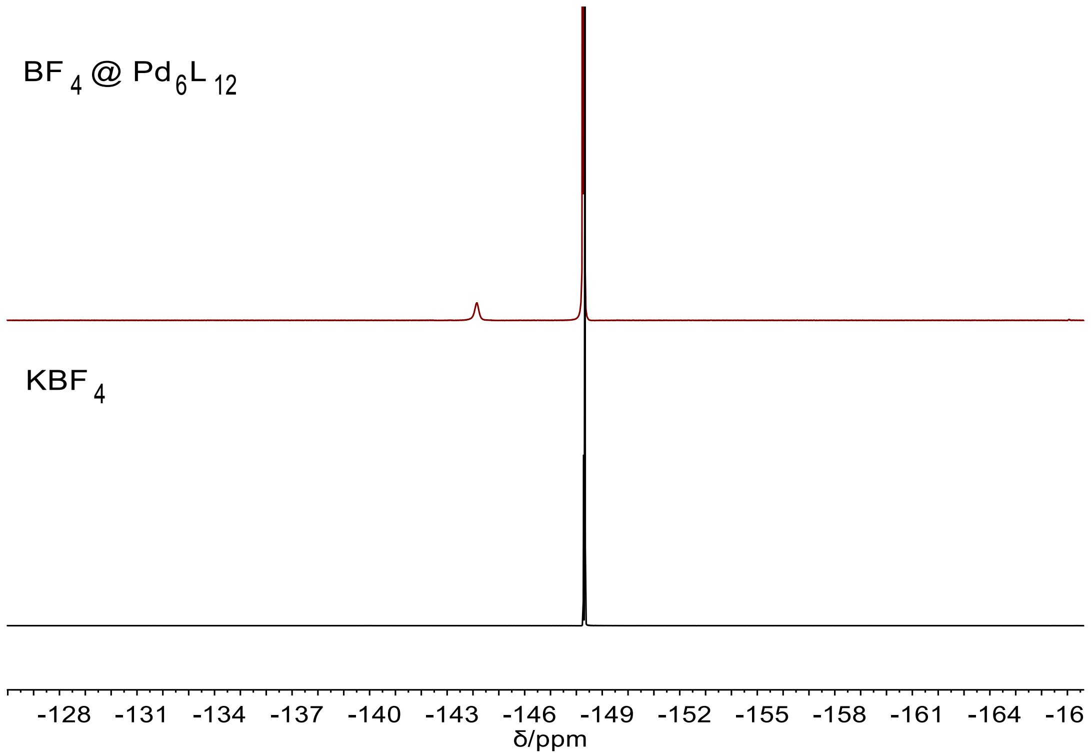

Fig. S21 ^{19}F NMR spectra of BF_4^-@Pd_6**L**$_{12}$ (top) and KBF_4 (down) in DMSO-d_6 (376 MHz, 298 K).

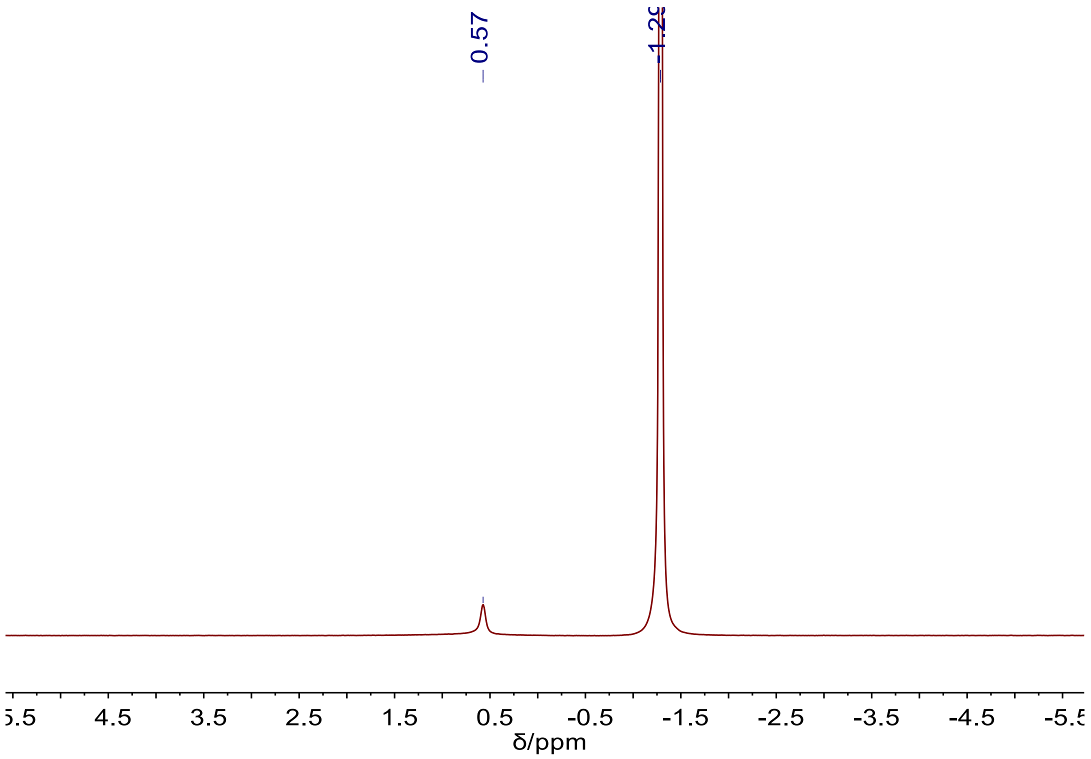

Fig. S22 ^{11}B NMR spectrum of BF_4^-@Pd_6**L**$_{12}$ in DMSO-d_6 (128 MHz, 298 K).

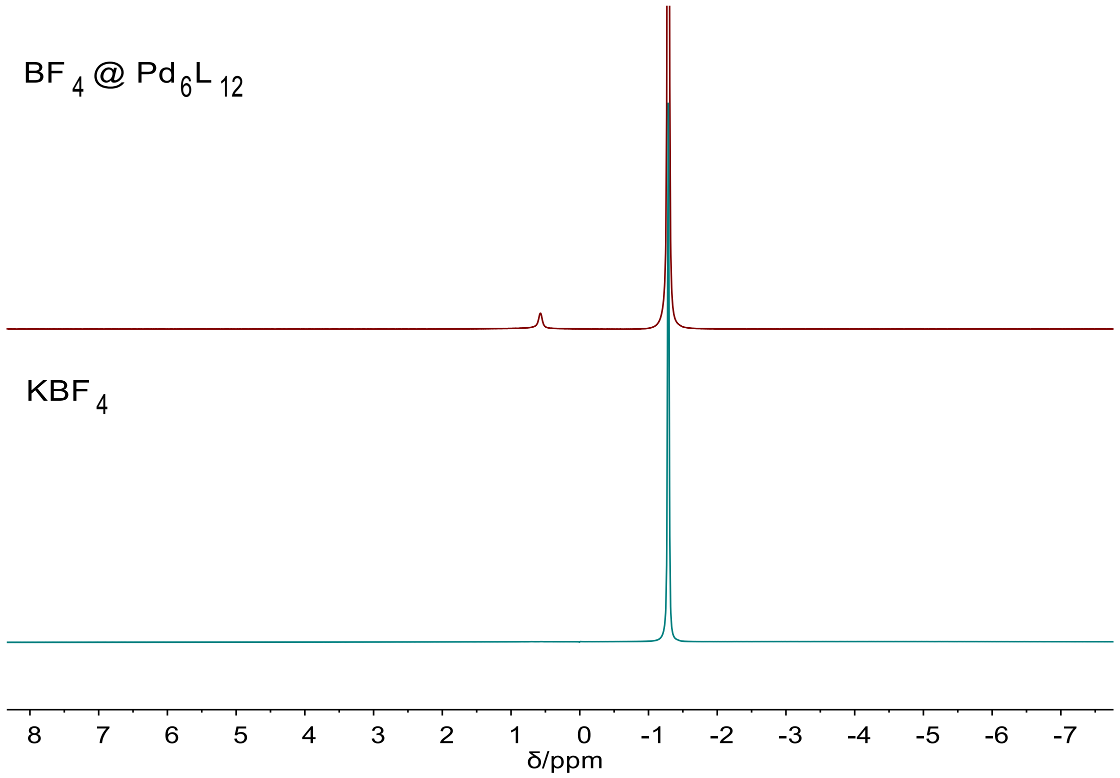

Fig. S23 ^{11}B NMR spectra of BF_4^-@$Pd_6\mathbf{L}_{12}$ (top) and KBF_4 (down) in DMSO-d_6 (128 MHz, 298 K).

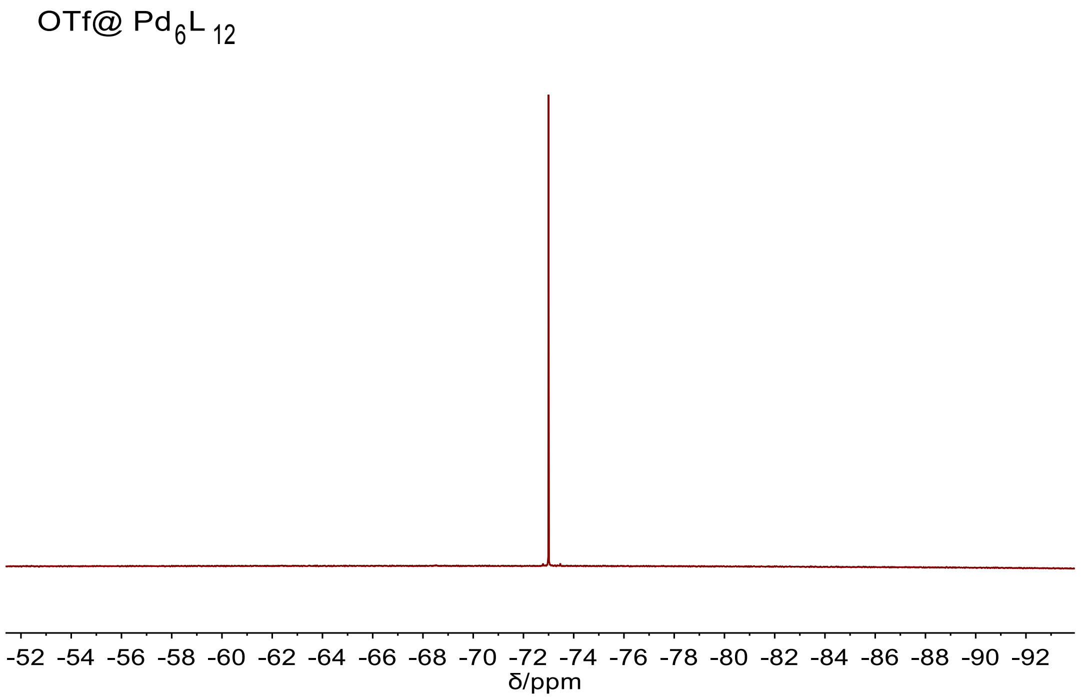

Fig. S24 ^{19}F NMR spectrum of OTf^-@$Pd_6\mathbf{L}_{12}$ in DMSO-d_6 (376 MHz, 298 K).

Table S1 Selected bond lengths (Å) and bond angles (˚) for $Pd_6\mathbf{L}_{12}$.

Au(1)-I(1)	2.488(4)	Pd(1)-N(4)	1.981(12)
Au(1)-C(18)	1.979(15)	Pd(1)-N(1)^1	1.99(2)
Au(2)-I(2)	2.514(3)	Pd(1)-N(5)	2.023(19)
Au(2)-C(55)	1.966(14)	Pd(1)-N(8)^2	1.96(12)
C(18)-Au(1)-I(1)	176.3(6)	N(4)-Pd(1)-N(1)^1	86.8(8)
C(55)-Au(2)-I(2)	179.4(5)	N(4)-Pd(1)-N(5)	176.8(7)
C(36)-N(4)-Pd(1)	119.7(9)	N(1)^1-Pd(1)-N(5)	90.9(9)
C(37)-N(4)-Pd(1)	119.9(9)	N(8)^2-Pd(1)-N(4)	90(6)
C(7BA)-N(1)-Pd(1)^3	118.5(15)	N(8)^2-Pd(1)-N(1)^1	176(5)
C(5)-N(1)-Pd(1)^3	119.9(16)	N(8)^2-Pd(1)-N(5)	93(6)

$^1 x - y + 1/3, x - 1/3, -z + 2/3$; $^2 -x + y + 1, -x + 1, z$; $^3 y + 1/3, -x + y + 2/3, -z + 2/3$; $^4 -y + 1, x - y, z$.
